# Advancing the prediction of bath penetration and electrochemical degradation in Hall-Héroult cell cathodes: Insights into ionic species transport in a porous electrode model

**DOI:** 10.1016/j.mex.2024.102593

**Published:** 2024-02-05

**Authors:** Yun Peng Zhang, Nan Zou, Shuang Jun Ma, Yang Youjian, Mouhamadou A. Diop

**Affiliations:** a School of Metallurgy, Northeastern University, Shenyang, 110819, PR China; b Key Laboratory for Ecological Metallurgy of Multimetallic Minerals (Ministry of Education), Shenyang, 110819, PR China

**Keywords:** Porous electrode model, Cathode, Degradation, Numerical simulations, Ionic species transport, Electroneutrality criterion, Finite Element Simulation of Hall-Héroult Cathode Degradation in Molten Salt

## Abstract

This study presents a groundbreaking approach to modeling the Hall-Héroult cathode used in aluminum production. Our innovative model is grounded in a sophisticated porous electrode methodology coupled with state-of-the-art numerical simulations. This enables us to capture the intricate physicochemical processes within the system precisely, encompassing the migration, diffusion, and convection of ionic species. A key feature of our model is the integration of detailed electrochemical reaction kinetics at the microscale, providing a nuanced understanding of the internal dynamics of the cathode. Furthermore, we have incorporated a unique penalization method that rigorously enforces the principles of electroneutrality and ionic thermodynamic equilibrium, ensuring the model's fidelity to real-world phenomena. Computational simulation using the finite element method (FEM) serves as the backbone of our model, offering unparalleled accuracy and robustness. This has been confirmed through validation against empirical data, underlining the model's potential to significantly enhance both the efficiency and sustainability of aluminum production processes.

• Development of an advanced porous electrode model for the Hall-Héroult process, utilizing numerical simulations42 to unravel complex physicochemical dynamics.
• Incorporation of a novel penalization method for ensuring electroneutrality and thermodynamic equilibrium, enhancing 44 model accuracy.
• Validation of the model against empirical data using FEM, demonstrating potential improvements in aluminum 46 production efficiency and sustainability.

## Highlights

• Introduces a novel model for analyzing physicochemical processes in aluminum production at the carbon pore level of Hall-Héroult cell cathodes.
• Focuses on the migration, diffusion, and convection of ionic species, alongside the electrochemical reactions crucial for aluminum production.
• Utilizes new numerical methods and the finite element method (FEM) for precise numerical calculations that align with experimental measurements.
• Aims to enhance the understanding and efficiency of aluminum production through detailed insights into ionic species transport in a porous electrode model.

## Graphical abstract

**Figure:**
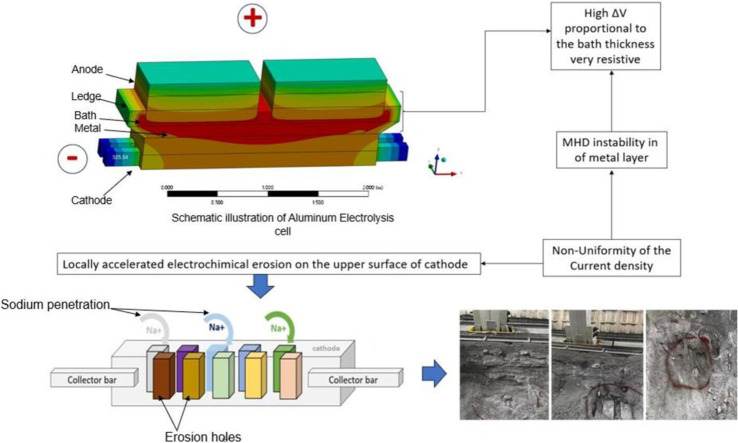

## Nomenclature

**Greek symbols**
ρ: density within a specific volume and sum of the product of species concentration Ci
ϕ: porosity of the solid medium
$\Psi_{sol}$: electrical potential of solid phase
ξ: tortuosity factor of the porous medium
$\rho^{sol}$: density of solid phase
$\rho_{\infty }$: reference density
$\Psi_{\text{liq}}$: electric potential in the liquid phase
$\xi_{\text{sol}}$: tortuosity factor for the solid phase
$\mu_{i}$: mobility of ion
κ: electric conductivity

**Roman symbols**
${\vec{j}}_{i}^{\text{liq}}$: flux of species i in liquid (mol.m-2s-1)
Ci: initial liquid phase concentration (mol.m-3)
$v_{i}^{liq}$: initial macroscopic Velocity of neutral molecules, ions or particles
${\vec{i}}^{liq}$: current density of liquid phase (A.m-2)
Zi: charge number of species i
F: faraday constant
Mi: molar mass of each species
b: empirical coefficient
x: mole fraction
$x_{\text{Al}F_{3}}$: mole fraction of AlF3
$x_{\text{NaF}}$: mole fraction of NaF
CR: concentration ratio
ni: number of moles within component i in the system
$n_{\text{NaF}}/V$: molar concentration of NaF ions
$n_{{\text{AlF}}_{4}^{-1}}^{\text{ion}}/V$: molar concentration of tetrafluoroaluminate ions
$n_{{\text{AlF}}_{6}^{3}}^{\text{ion}}/V$: molar concentration of hexafluoroaluminate ions
$C_{Na^{+}}$: ionic concentration of Sodium specie
$C_{{\text{AlF}}_{4}^{-1}}$: ionic concentration of ${\text{AlF}}_{4}^{-1}$specie
$C_{{\text{AlF}}_{6}^{-3}}$: ionic concentration of ${\text{AlF}}_{6}^{-3}$specie
Subscript i: component I within the system
D: diffusion coefficient
${\bar{D}}_{i}$: effective diffusion coefficient for ion i
$x_{NaF}^{bin}$: binary mole fraction of NaF
$x_{\text{Al}F_{3}}^{\text{bin}}$: binary mole fraction of AlF3
${\vec{v}}^{\text{liq}}$: velocity vector field
S: entropy or source term, depending on context
μ: mobility of ion
κ: electrical conductivity
q: parameter related to pore structure
T: temperature
P: scalar potential
U: potential difference
R: universal gas constant

Specifications table

**Table:** 

Subject area:	Materials Science
More specific subject area:	Aluminum Electrolysis Process
Name of your method:	Finite Element Simulation of Hall-Héroult Cathode Degradation in Molten Salt
Name and reference of the original method:	F. Gagnon, D. Ziegler, & M.Fafard, Electrochemical modelling using electroneutrality equation as a constraint, J.Appl.Electrochem., 44(3) (2014)361–381. https://doi:10.1007/s10800-014-0662-6.
Resource availability:	N.A.

### Introduction

In the realm of modern technological advancements, aluminum emerges as a quintessential material, celebrated for its remarkable contributions to energy efficiency and sustainability. The allure of this metal lies in its trifecta of advantageous properties: a unique blend of lightweight yet robust mechanical strength, commendable resistance to corrosion, and exceptional electrical and thermal conductivity. These intrinsic qualities have propelled aluminum to the forefront of diverse industrial applications, particularly in sectors dedicated to environmental preservation and resource efficiency. Evidencing this trend, global aluminum production surged to an impressive 67.343 million tons in 2021, marking a significant increase of 10.9 % since 2015 [1]. Despite substantial advancements in aluminum recycling and remelting techniques, primary aluminum production continues to play a pivotal role in meeting industry demands [2].

At the heart of primary aluminum production stands the Hall-Héroult process, renowned for its pivotal role and energy-intensive nature. Notably, approximately 60 % of the world's aluminum production in 2020 relied on hydroelectric power sources, showcasing the industry's commitment to sustainability [3]. This intricate process revolves around the electrolysis of alumina, primarily sourced from bauxite ores via the Bayer process, within a cryolite-based solution. The operation of electrolysis cells, characterized by a carbon anode and a metallic cathode, poses inherent challenges, including forming alumina-cryolite sludge and releasing gaseous by-products, significantly influencing the cell's efficiency and longevity.

The operational lifecycle of an electrolytic cell encompasses various stages, from preheating to the commencement of electrolysis, ultimately leading to full operational capacity. These stages are marked by a complex interplay of physical and chemical transformations, each bearing profound implications for the cell's performance. Among these, the degradation of the cathode emerges as a pivotal focal point, driven by a myriad of mechanical and chemical factors. This degradation carries substantial environmental and economic consequences, underlining the imperative need for predictive models to comprehend and preemptively address these intricate processes.

This study represents a pioneering effort in modeling the dynamic behavior of cathodes within the Hall-Héroult cell, providing valuable insights into the complex phenomena that transpire during the initial phases of aluminum electrolysis cells. Our innovative approach seamlessly integrates advanced ionic transport laws and leverages state-of-the-art numerical techniques, as described in [4], [5], [6], tailored to simulate the multifaceted chemical and electrochemical processes at play. The essence of innovation in this research lies in its comprehensive methodology, shedding new light on the mechanisms governing cathode degradation and significantly advancing the field of aluminum production.

The novelty of our approach is underscored by its multifaceted exploration of the dynamics within the electrolysis cell, with a particular emphasis on understanding and modeling the behavior of the cathode. This endeavor encompasses the incorporation of a sophisticated ion formulation harmonized with a transient porous electrode model, complemented by a novel technique for maintaining electroneutrality. This study represents a substantial leap in comprehending cathode degradation mechanisms and opens up vast avenues for future research. These potential extensions of our work hold promise in the realms of predictive modeling for cathode lifespans, optimization of operational conditions, and the development of more efficient and enduring cathodes, with practical applications spanning the aluminum industry and high-temperature electrolytic processes. Our contributions, pivotal not only for the aluminum industry but also as a benchmark for research in analogous high-temperature electrolytic processes, underscore the significance and ingenuity of this scientific investigation [7,8].

### Method details

#### Fluid-solid coupling model

##### Gas-Liquid flow model

This section describes the charge and species conservation equations that help elaborate the porous electrode model [9]. The equations are described in a general way without defining the constitutive relations. The equations are defined for the liquid phase of the cryolite bath and for the porous carbon cathode whose pores are impregnated with the same liquid phase. Subsequently, the constitutive species transport equations are defined for the porous and non-porous medium. First, the definitions of fluxes in the liquid phase must be presented to explain some simplifying assumptions of the model. The molar flux of a species [9,10] in the liquid phase of concentration c_i_ (mol.m^−3^) is defined as follows:

(1) $${\vec{j}}_{i}^{liq}=c_{i}{\vec{v}}_{i}^{liq}$$

${\vec{v}}_{i}^{\text{liq}}$ denotes the macroscopic speed of neutral molecules, ions, or colloid particles moving with the liquid phase.

Subsequently, the flow of a species can be defined from a reference velocity of the liquid phase [9]:

(2) $${\vec{J}}_{i}^{liq}={\vec{j}}_{i}^{liq}-c_{i}{\vec{v}}^{liq}$$

Several types of reference speeds can be chosen [10]. In this study's context, the velocity used above as a referee is the barycentric one and states [10]

(3) $${\vec{v}}^{liq}=\frac{1}{\rho^{liq}}\sum_{i}\rho_{i}^{liq}{\vec{v}}_{i}^{liq}$$

This choice of reference velocity is usually more adequate for modeling fluid flow. From the previous definitions of fluxes, we can define the current density for the liquid phase [7,9,10]:

(4) $${\vec{i}}_{i}^{liq}=F\sum_{i}z_{i}{\vec{J}}_{i}^{liq}$$

For the systems studied, the electroneutrality criterion will be considered valid throughout the modeled domain [9],

(5) $$\sum_{i}z_{i}c_{i}=0$$

The electroneutrality hypothesis results in the absence of a current density of a convective nature, as shown by the studies of [11]. This becomes evident by substituting Eq. (2) in Eq. (4) and using the electroneutrality relation. The absence of a convective source current does not imply the absence of convection. To model the convection due to the ionic transport phenomena, a numerical scheme based on mass conservation is used [9], [10], [11], and it writes as follows:

(6) $$\frac{\partial \rho }{\partial t}=-\nabla \cdot \left(\rho {\vec{v}}^{liq}\right)$$

In the above-mentioned mass conservation in Eq. (6), the density can be defined as a function of the molar mass Mi and the species concentration c_i_ by the following expression [10,11]:

(7) $$\rho =\sum_{i}c_{i}M_{i}$$

It is possible to use an empirical relation depending on the concentration of the ions to give the density. In the model case, the observed relationship of Ratvik et al. [12] without alumina and additives will be simplified and used for the density as stipulated in [10], [11], [12], and it is written as follows:

(8) $$\rho_{T}=\rho_{1000}-b\left(T-1000\right)$$

With,

(9) $$\rho_{1000}={\left[{\left(1946+1113x_{AlF_{3}}^{bin}\right)}^{-3.6}+{\left(\left(x_{AlF_{3}}^{bin}\right)/859\right)}^{3.6}\right]}^{-1/3.6}$$

And,

(10) $$b=0.64x_{NaF}^{bin}+\frac{1.7x_{AlF_{3}}^{bin}+1.5{\left(x_{AlF_{3}}^{bin}\right)}^{2}}{1+140x_{NaF}^{bin}\cdot {\left(x_{AlF_{3}}^{bin}\right)}^{5}}$$

T stands for the temperature in ° C; the mole fractions expressed in terms of NaF and AlF_3_ for the NaF-NaAlF_4—_Na_3_AlF_6_ system are implemented in Eqs. (9) and (10).$x_{\text{NaF}}^{\text{bin}}$ et $x_{\text{Al}F_{3}}^{\text{bin}}$ are the fractions molaires obtenues pour les différentes valeurs de CR.

The combination of Eqs. (8)–(10) depicts an empirical relationship for calculating the density (ρ_liq_) of a solution in an electrochemical model, which is dependent on the concentration of ions and temperature. The density is given by Eq. (8), which is a function of temperature and a term 'b', subtracted from a reference density ρ∞. The reference density ρ_∞_ is defined by Eq. (9), which includes ionic fractions and an empirical exponent. The term 'b' from Eq. (10) is calculated using the ionic fractions of NaF and AlF3. This relationship is simplified from a form provided by Ratvik et al. for use in the model without alumina and additives.

The molar concentrations of ions for a given CR are obtained with the following equation Eq. (11) as stated in [9,11]:

(11) $$\left\{ \begin{array}{c} CR=\frac{x_{NaF}^{bin}}{x_{AlF_{3}}^{bin}}\\ x_{NaF}^{bin}+x_{AlF_{3}}^{bin}=1 \end{array}\right.$$

Assuming a full ionization of the species in the system mentioned above, the n_i_ values come from the following equation,

(12) $$x_{NaF}=1-x_{NaAlF_{4}}-x_{NaAlF_{6}}$$

Finally, one obtains the numbers of moles for the molar fractions of the system species , which writes as:

(13) $$n_{i}=Nx_{i}$$

So, for the ions F^−1^, Na^+1^,${\text{AlF}}_{4}^{-1}$, and${\text{AlF}}_{6}^{-3}$, the number of moles versus volume for a given CR is given by:

(14) $$\left\{ \begin{array}{c} n_{F^{-1}}^{ion}=\frac{n_{NaF}}{V}\\ n_{Na^{+}}^{ion}=\frac{n_{NaF}+n_{NaAlF_{4}}+3n_{Na_{3}AlF_{6}}}{V}\\ n_{AlF_{4}^{-1}}^{ion}=\frac{n_{NaAlF_{4}}}{V}\\ n_{AlF_{6}^{-3}}^{ion}=\frac{n_{Na_{3}AlF_{6}}}{V} \end{array}\right.$$

In order to ensure the consistency of the procedure, the CR can be recalculated with the following equation:

(15) $$CR=\frac{c_{Na^{+}}}{c_{AlF_{4}^{-1}}+c_{AlF_{6}^{-3}}}$$

Similarly, the CR can be written as a function of the ionic concentrations of the species $c_{Na^{+1}}$,$c_{{\text{AlF}}_{4}^{-1}}$, and $c_{{\text{AlF}}_{6}^{-3}}$ (see Eq. (15)). So, when taking the constant temperature with the chain rule, the time and space derivatives of the empirical equation describing the density (Eq. (8)) are given as follows:

(16) $$\frac{\partial \rho_{T}}{\partial t}=\left[\left(\frac{\partial \rho_{1000}}{\partial x_{NaF}^{bin}}-\frac{\partial b}{\partial x_{NaF}^{bin}}\left(T-1000\right)\right)\frac{\partial x_{NaF}^{bin}}{\partial CR}+\left(\frac{\partial \rho_{1000}}{\partial x_{AlF_{3}}^{bin}}-\frac{\partial b}{\partial x_{AlF_{3}}^{bin}}\left(T-1000\right)\right)\frac{\partial x_{AlF_{3}}^{bin}}{\partial CR}\right]\frac{\partial CR}{\partial t}$$

The above equation seems to apply the chain rule to relate the partial derivatives of density with respect to time and CR, incorporating temperature as a constant. This type of calculation is often used in the context of electrochemistry in molten salt, where understanding how properties like density change with composition and time is crucial for process control and materials characterization.

Finally, the gradient of density was retrieved using a more elaborated expression as in [7,[9], [10], [11], [12]] and written as in Eq. (17):

(17) $$\nabla \rho_{T}=\left[\left(\frac{\partial \rho_{1000}}{\partial x_{NaF}^{bin}}-\frac{\partial b}{\partial x_{NaF}^{bin}}\left(T-1000\right)\right)\frac{\partial x_{NaF}^{bin}}{\partial CR}+\left(\frac{\partial \rho_{1000}}{\partial x_{AlF_{3}}^{bin}}-\frac{\partial b}{\partial x_{AlF_{3}}^{bin}}\left(T-1000\right)\right)\frac{\partial x_{AlF_{3}}^{bin}}{\partial CR}\right]\nabla CR$$

Eq. (17) denotes a gradient operation applied to an empirical density function ρ_liq_ in terms of the concentration ratio (CR) and temperature (T). This equation likely arises from a complex model where the density of a liquid phase in a process (such as in electrolytic cells) depends on the concentration of species and temperature. It represents a mathematical way to express how density changes spatially within the system, accounting for the effects of concentration changes and temperature, which are crucial for simulations and process optimizations.

The different derivatives are present in the above equations. Eqs. (6)–(9) will give the following numerical expressions:

(18) $$\frac{\partial \rho_{1000}}{\partial x_{NaF}^{bin}}=0$$

(19) $$\frac{\partial b}{\partial x_{NaF}^{bin}}=0.64-\frac{\left(1.7x_{AlF_{3}}^{bin}+1.5{\left(x_{AlF_{3}}^{bin}\right)}^{2}\right)\left(140\cdot {\left(x_{AlF_{3}}^{bin}\right)}^{5}\right)}{{\left(1+140x_{NaF}^{bin}\cdot {\left(x_{AlF_{3}}^{bin}\right)}^{5}\right)}^{2}}$$

(20) $$\begin{array}{ccc} \frac{\partial \rho_{1000}}{\partial x_{AlF_{3}}^{bin}} & = & \frac{-1}{3.6}\left[{\left(1946+1113x_{AlF_{3}}^{bin}\right)}^{-3.6}\right]\left(\frac{-4.6}{3.6}\right)\\ & & \left[-4006{\left(1946+1113x_{AlF_{3}}^{bin}\right)}^{-4.6}+\frac{3.6}{859}{\left(x_{AlF_{3}}^{bin}/859\right)}^{2.6}\right] \end{array}$$

(21) $$\frac{\partial b}{\partial x_{AlF_{3}}^{bin}}=\frac{1.7+3x_{AlF_{3}}^{bin}}{1+140x_{NaF}^{bin}\cdot {\left(x_{AlF_{3}}^{bin}\right)}^{5}}-\frac{\left(1.7x_{AlF_{3}}^{bin}+1.5{\left(x_{AlF_{3}}^{bin}\right)}^{2}\right)\left(700x_{NaF}^{bin}\cdot {\left(x_{AlF_{3}}^{bin}\right)}^{4}\right)}{{\left(1+140x_{{}_{NaF}}^{bin}\cdot {\left(x_{AlF_{3}}^{bin}\right)}^{5}\right)}^{2}}$$

(22) $$\frac{\partial x_{NaF}^{bin}}{\partial CR}=\frac{1}{1+CR}-\frac{CR}{{\left(1+CR\right)}^{2}}$$

(23) $$\frac{\partial x_{NaF}^{bin}}{\partial CR}=-\frac{1}{{\left(1+CR\right)}^{2}}$$

(24) $$\frac{\partial CR}{\partial t}=\frac{1}{c_{AlF_{6}^{-3}}+c_{AlF_{4}^{-1}}}\left[\frac{\partial c_{Na^{+}}}{\partial t}\right]-\frac{c_{Na^{+}}}{c_{AlF_{6}^{-3}}+c_{AlF_{4}^{-1}}}\left[\frac{\partial c_{AlF_{4}^{-1}}}{\partial t}+\frac{\partial c_{AlF_{6}^{-3}}}{\partial t}\right]$$

(25) $$\nabla CR=\frac{1}{c_{AlF_{6}^{-3}}+c_{AlF_{4}^{-1}}}\left[\nabla c_{Na^{+}}\right]-\frac{c_{Na^{+}}}{c_{AlF_{6}^{-3}}+c_{AlF_{4}^{-1}}}\left[\nabla c_{AlF_{4}^{-1}}+\nabla c_{AlF_{6}^{-3}}\right]$$

The above differential equations and expressions are likely derived from a set of physicochemical principles related to a specific research context. These equations detail how different parameters and concentrations vary with respect to each other and time. They are mathematical representations of the behavior of a system under study, possibly involving concentration ratios, partial derivatives as a function of time, and other related variables. This type of detailed mathematical modeling is crucial in electrochemistry, such as molten salt, where understanding the dynamic changes in a system is essential for process control, design, and optimization.

Now, one can return to the expression of the density as a function of molar mass in Eq. (7) and simplify the mass conservation model. Thus, the velocity vector field ${\vec{v}}^{\text{liq}}$ is considered conservative, making it possible to define the speed as a function of a potential P. Thus, one obtains this below expression as demonstrated in [8,10]:

(26) $${\vec{v}}^{liq}=-\nabla P$$

So, the convection velocity is obtained by Eq. (26), but by solving the conservation equation in the following form:

(27) $$\frac{\partial \rho }{\partial t}=-\nabla \cdot \left[-\rho \nabla P\right]$$

Eq. (27) is only corrected for porosity in a medium like a cathode block. Dispersion effects are neglected [11]. The mass conservation equation for the porous medium is written as follows:

(28) $$\phi \frac{\partial \rho }{\partial t}=-\nabla \cdot \left[-\phi \rho \nabla P\right]$$

It should be noted that the velocity of the porous solid medium is zero. It is thus possible to develop a more complex flow model. Still, in the context of this study, it was considered less important than the development of aspects of ion transport and the implementation of electrochemical reactions.

##### Liquid phase conservation equations

In the cryolite bath supernatant, the cathode is defined as a simple liquid phase at a constant temperature of about 960 °C. The species conservation equation for the liquid phase writes [6,9,11]:

(29) $$\frac{\partial c_{i}}{\partial t}+\nabla \cdot \left({\vec{J}}_{i}^{liq}\right)-S_{i}=0$$

Where S_i_ represents a source of species from a homogeneous chemical reaction.

Thus, the charge conservation equation for the liquid phase is obtained and writes in this form:

(30) $$\nabla \cdot {\vec{i}}^{liq}=0$$

The transport of ionic species in the cryolite bath is described using formulations taken from [10], [11], [12], [13], [14], [15] and which are written as follows,

(31) $${\vec{J}}_{i}^{liq}=-D_{i}\nabla c_{i}-z_{i}c_{i}Fu_{i}\nabla \psi_{liq}+c_{i}{\vec{v}}^{liq}$$

This above Eq. (31) details the ionic flux, combining effects of diffusion, electric field influence, and convection. Therefore, this study considered that the transport of ionic species is essentially through the forces associated with the concentration and potential gradients of the cryolite bath. Therefore, the Nernst-Einstein equation is assumed to be valid in molten salts [16], and one can write:

(32) $$D_{i}=STu_{i}$$

Thus, the Nernst-Einstein relation (Eq. (32)) connects ionic mobility to diffusion coefficients, emphasizing its validity in molten salts, which is fundamental in modeling the electrolytic processes in aluminum smelting.

Diffusion and migration parameters were calculated from experimental conductivity data. Ohm's law is respected as part of the measurements used for calibration, which means that the concentration gradients are zero. Thus, for the bath this used Ohm's law [7,11,13] writes as shown below:

(33) $${\vec{i}}^{liq}=-\kappa^{liq}\nabla \psi_{liq}$$

In this context, Ohm's law is applied assuming that the concentration gradients are zero, simplifying the expression for current density in the electrolyte. Eq. (33) is then presented as a formulation of Ohm's law to describe the current in terms of conductivity and the electric potential gradient in the liquid phase. This is fundamental for calibrating and validating the electrochemical model being described.

Thus, by using the zero-concentration gradient assumption and combining Eqs. (4), (12), (17)–(19), we obtain the relationship between the diffusion coefficients and the conductivity [9,11,13]:

(34) $$\kappa^{liq}=F^{2}\sum_{i}\frac{z_{i}^{2}c_{i}D_{i}}{ST}$$

The diffusion coefficients are independent of the wedging, similar to a crystalline solid [17]. As mentioned above, according to the scale of the problem, the current transport by convection is zero for a system called an electrometer [11] macroscopically. Eq. (34) combines these ideas to relate the diffusion coefficients with the system's conductivity. This relationship is crucial for understanding how ions move within the electrolyte and affect the cell's overall efficiency and performance.

#### Porous cathode block

Diffusion coefficients and ion mobilities must be corrected to consider the porous medium's effect. The effect of tortuosity defines the effect of the porous medium, i.e., the diffusion of species in the liquid phase cannot occur in a straight line but must follow the tortuous paths connecting the constituent porosities of the solid. The Nernst-Einstein equation is assumed to be valid, and the ion mobility is corrected in the same way for the porosity effect as for the diffusion of a neutral species, so there is no difference for the diffusion or mobility case versus the effect of the loaded walls. Only the dissolved metal could be considered neutral, but the latter is supposed to be affected by the electric field [18], [19], [20], [21], [22], [23], [24], [25], [26]. For an identical correction for the effect of tortuosity, the Nernst-Einstein equation was used with the diffusion coefficients, and the corrected expression of the ion mobilities as given in [8], [9], [10], [11], [12], [13] will be given by:

(35) $${\bar{D}}_{i}=ST{\bar{u}}_{i}$$

The hypothesis of neglecting the effect of the charged walls remains valid if the electrostatic effect of the latter does not locally affect the transport between the pores and the cavities. Therefore, this assumption becomes invalid in a system with narrow pores and highly charged walls. The tortuosity correction is not necessarily the same for neutral species diffusion coefficients compared to ion mobility. Thus, the correction is unrelated to the Nernst-Einstein equation, and the equality of the latter remains accurate and independent of the porosity. The chosen tortuosity model is borrowed from a relationship for a packed-bed type chemical reactor described by Pollard et al. in [27,28] and Whitaker [29] and writes as follows:

(36) $$\varsigma =\phi^{\left(1-q\right)/2}$$

This model of tortuosity is a function of the porosity ϕ and the value of the parameter, which is fixed at 1.5 as indicated in [28], which gives a corrected expression of the classical tortuosity equation:

(37) $$\varsigma =\phi^{-0.25}$$

Another more complex tortuosity model could have been chosen, but y this was not deemed necessary for simplicity. Thus, the development of a more appropriate model from measurements on industrial cathode blocks will be the logical next step to refine the numerical calculations.

On the other hand, ${\vec{J}}_{i}^{\text{sup}}$the surface flow formulation of Newman et al. [11] in a porous medium for the species in the liquid phase impregnating the cathodic pores is obtained by combining the transport parameters corrected for the tortuosity with the contribution of the porosity, which will give an adequate expression which is written as follows:

(38) $${\vec{J}}_{i}^{\sup}=-\frac{D_{i}}{\varsigma^{2}}\nabla \left(\phi c_{i}\right)-z_{i}\phi c_{i}F\frac{u_{i}}{\varsigma^{2}}\nabla \psi_{liq}+\phi c_{i}{\vec{v}}^{liq}$$

In the context of the Hall-Héroult process, it could be an equation that relates to the flow of ions in a porous medium, accounting for factors like porosity and tortuosity as they impact the transport phenomena within the cathodic structure.As it should be noted that the concentration ci is considered for the liquid and not for the porous solid domain, hence the necessary inclusion of the porosity in Eq. (38) to define surface flux according to the homogenization theories [10]. Thus, the porosity taken as a constant in Eq. (38) can be simplified by including tortuosity to take the following form [10]:

(39) $${\vec{J}}_{i}^{\sup}=-D_{i}\phi^{1.5}\nabla c_{i}-z_{i}c_{i}Fu_{i}\phi^{1.5}\nabla \psi_{liq}+\phi c_{i}{\vec{v}}^{liq}$$

These equations describe the transport parameters for species within a porous medium, specifically in the context of the cathode used in the Hall-Héroult process. It details how to account for porosity when determining the surface flow of ions within the cathodic pores. The equations shown are adaptations of the classical Nernst-Planck equation, adjusted for the porosity and tortuosity of the medium. These corrections are essential for accurately modeling the flux of ions, as they navigate the complex pathway within the porous structure and the electronic conduction of the cathode block itself, which is influenced by these transport parameters.

Therefore, the cathode block's electronic conduction depends on Ohm's law. The same principle of correction for tortuosity is applied to the carbon conductivity$\kappa_{Sol}$; and it is assumed that the tortuosity effect for a fraction of solid is reciprocal to the tortuosity effect associated with the pores which allow us to use the below equation:

(40) $$\varsigma_{sol}={\left(1-\phi \right)}^{\left(1-q\right)/2}$$

From these modifications and the usage of the porosity, the surface current density equation was retrieved for the solid domain as stipulated in [7,8,11,13,14]

(41) $${\vec{i}}^{sol}=-\left(1-\phi \right)\frac{\kappa_{sol}}{\varsigma_{sol}^{2}}\nabla \psi_{sol}$$

#### Validation model and boundary conditions

**Fig. 1:**
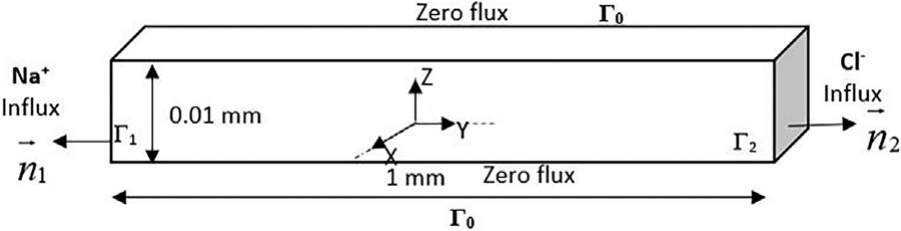
Geometry of the conduction system in an aqueous medium through a pore channel.

To conduct a comparative analysis for implementing electroneutrality through various methods, this study utilizes a conductivity problem in an aqueous medium, as outlined in the research of Sarkar et al. [27]. The problem to be solved is a simple transient ionic conduction considered brine in which the charge carrier is salt (NaCl) completely dissociated in water. The solution is dilute and stagnant, making the convection negligible. The current imposed at the boundaries is constant. It comes from a molar flux of Na^+^at one of the boundaries and an electrically equivalent Cl^−^ flux at the other boundary. The system does not allow a free net charge, so it must remain electrically neutral macroscopically. One assumes that there is no source term. Fig. 1 below shows the spatial resolution domain. Table 1 presents the initial and boundary conditions of the problem as well as the diffusion coefficients. The mobilities u_i_ are obtained from the Nernst-Einstein equation (Eq. (35)). It should be noted that the initial and boundary conditions on the Cl^−^ion and the Lagrangian variable λ do not apply in the case of the method eliminating the Cl^−^ species conservation equation.

**Table 1:** Conditions and parameters of the conduction problem at T = 298.15 K.

Diffusion Coefficients and Ion Charge				
T	D_N_a+	Z_Na_^+^	D_Cl_^−^	Z_Cl_^−^
298.15 K	1.33 × 10^−9^ m^2^.s^−1^	1	2.03 × 10^−9^ m^2^.s^−1^	−1

Initial Conditions				

c_Na_^+^(x,0)	c_Cl_^−^(x,0)	Ф_liq_(x,0)	λ(x,0)	

0.01 mol.m^−3^	0.01 mol.m^−3^	0V	1	

Boundary Conditions				

Γ_1_	Γ_2_	Γ_0_		

$-\vec{n}\times {\vec{J}}_{cl^{-}}=0$ mol.m^2^s^−1^	$-\vec{n}\times {\vec{J}}_{cl^{-}}=0$ mol.m^2^s^−1^	$-\vec{n}\cdot {\vec{J}}_{cl^{-}}=0$ mol.m^2^s^−1^		
$-\vec{n}\times {\vec{J}}_{cl^{-}}=1\times 10^{-6}$ mol.m^2^s^−1^				
$-\vec{n}\cdot i=Fz_{Na^{+}}J_{Na^{+}}$	$-{\vec{n}}_{{}_{2}}\cdot i=Fz_{cl^{-}}J_{cl^{-}}$			

#### Mesh conditions

Table 2 presents the different parameters utilized in the numerical finite element resolution of COMSOL Multiphysics 6.0 (see supplementary Tables S1–S4). The parameters for the mesh and equation solutions are identical, with the exception of the specified application mode in the Table. It is important to mention that the mesh used is symmetrical.

**Table 2:** Model mesh parameters.

Parameters	Values		
Consistent initialization of diff./algebraic equations	Backward Euler		
Implicit method with variable order and variable time step	BDF (Backward differentiation formula)		
Time interval	t∈[0,45 s]		
Direct linear solver	UMFPACK		
Relative tolerance	10^−3^		
Absolute tolerance	10^−4^		
Meshing type	Free mesh		
Meshing method	Triangle Advancing Front		
Meshing dimension	Extra Fine		
Meshing refinement method	Regular		
Number of triangular elements	9600		

Parameters	Simple method	Lagrange constraint method	COMSOL 6.0

Number of freedom degrees	10,786	21,572	10,786
Application mode	PDE general form	PDE general form	Nernst-Planck

#### Comparison of different methods for the electroneutrality implementation

**Fig. 2:**
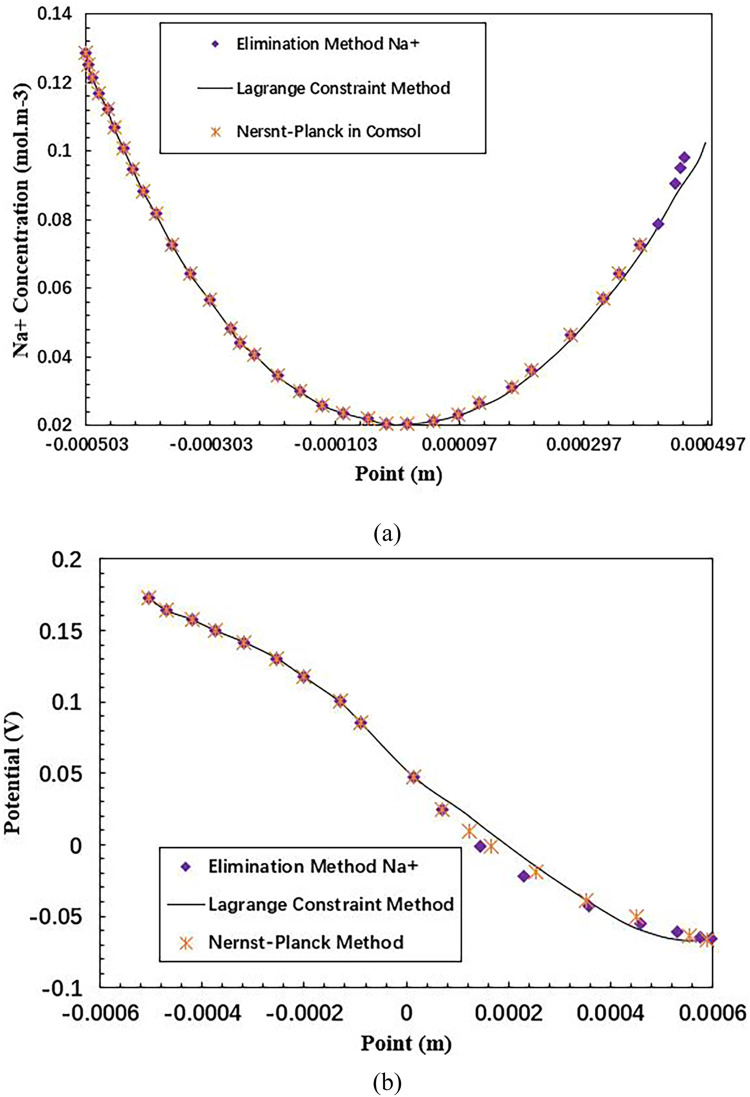
Evolution of the Na^+^concentration and potential in the aqueous solution as a function of the point at time *t* = 60 s : (a) Na^+^ concentration ; (b) Potential.

In Fig. 2 (a), the concentration profile of Na^+^ species at 60 s is shown for two electroneutrality implantation methods, as well as the results obtained from the Nernst-Planck application mode of the finite element software COMSOL Multiphysics 6.0. The curves obtained from all three methods are identical, producing consistent results. The profiles of Cl^−^ ion are not shown because they are identical to those of the Na^+^ion.

Similarly, in Fig. 2 (b), the potential curves at 60 s are shown, and they are found to be similar for both electroneutrality implantation methods and the Nernst-Planck application mode. This also indicates that the methods used are producing consistent and accurate results (see supplementary Tables S2–S4).

**Fig. 3:**
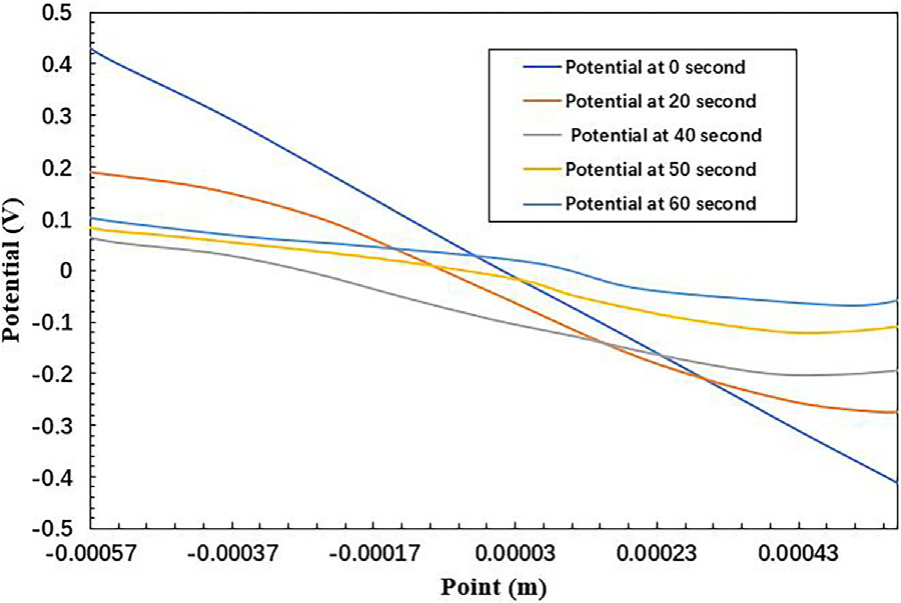
Evolution of the aqueous solution's potential as a time function.

In Fig. 3, the potential is shown as a function of position and time. The potential profiles are consistent with what is expected, with areas of higher conductivity (due to higher concentrations of NaCl) showing a lower potential drop. These results are similar to those obtained by Sarkar et al. [30] for concentration profiles but different for the potential. The difference is due to the fact that Sarkar et al. used Laplace's equation to solve the problem, which assumes that the conductivity in the aqueous medium is constant as a function of position at each time step, resulting in linear potential curves. In contrast, the method used in this section takes into account the changing conductivity as a function of position and time, resulting in non-linear potential curves.

Table 3 shows the performances in respect of electroneutrality (see supplementary Table S5). The results are similar. The error on the Lagrange multiplier type method is of the order of the machine error 2.2x l0×6 (algorithmic method [[24], [31]]) evaluated for the computer used in the calculations. Therefore, applying electroneutrality by Lagrange constraint produces results consistent with the other methods considered in this study (see supplementary Figs. S1 and S2).

**Table 3:** Verification of electroneutrality on the resolution domain at 60 s.

Methods	Electroneutrality $\sum_{i}z_{i}c_{i},\;i=Na^{+},Cl^{-}$
Elimination method	0
Lagrange type of constraint	[−5.6 × 10^−17^, 4.2 × 10^−17^]
Nernst-Planck in COMSOL 6.0 Multiphysics	0

The system studied consists of a porous cathode and a cryolite bath resting on the cathode. The porous cathode consists of a conductive solid of a graphitic nature whose pores are impregnated with a liquid phase composed of molten fluorides (NaF and AlF_3_). The liquid phase that submerges the porous cathode consists of the same liquid phase that occupies the cathode's pores. The liquid phase is continuous between the cathode and the medium external to the cathode. The cathode block is defined as continuous with a homogenization method for a porous medium. The electrochemical reaction of aluminum production in the porous domain makes the electrical link between the solid and liquid phases. The metal produced is considered to dissolve instantly and not affect the properties of the medium. The system is transient and maintained at 1026.85° C. This isothermal system involves diffusion, migration, and convection transport in the liquid phase. The conduction in the solid phase is of electronic type only.

The partial differential equations, constitutive laws, parameters, and various associated assumptions have been presented in detail in Sections 2.1–2.3. In this section, only the final assembled equations are presented. The equation system consists of five main equations for species, two for potential evolution, and the mass conservation equation. The equations are given in strong forms with weights. Eqs. (42) and (43) are the conservation equations for ionic species. Eq. (42) is defined for the liquid phase of the non-porous domain, while Eq. (43) is for the same liquid phase present in the porous medium (see supplementary Tables S1 & S2) and as demonstrated in [3,7,8,10,11]:

(42) $$\int \sum_{i}\delta c_{i}\left[\frac{\partial c_{i}}{\partial t}+\nabla \cdot \left(-D_{i}c_{i}-z_{i}c_{i}Fu_{i}\nabla \phi_{liq}-c_{i}\nabla \Pi \right)-k\psi_{i}s_{i}\left[K-\frac{a_{NaF}^{2}c_{AlF_{4}^{-1}}}{c_{AlF_{6}^{-3}}}\right]+z_{i}\lambda \right]dV=0i=Na^{+},F^{-1},{\text{AlF}}_{4}^{-1},AlF_{6}^{-3},Al^{0}$$

The above Eq. (42) is a species conservation equation for the liquid phase in a non-porous domain. This type of equation typically includes the accumulation of species, advection, diffusion (Fick's law), and source terms due to chemical reactions. The equation sums all species i and takes into account the changes in concentration of species i with time, the velocity field of the liquid phase, the diffusion coefficient for each species, and the electric field effect on ionic species. It also includes a reaction term that represents the rate of generation or consumption of species i due to chemical reactions. This equation is integral to modeling the behavior of electrolytes in electrochemical systems like the Hall-Héroult cell.

The conservation equation for ionic species within the liquid phase present in the porous medium is given by Eq. (43). This equation accounts for multiple phenomena: the time rate of change of ionic concentration, the advection of ions through the medium, the diffusion of ions under the influence of a concentration gradient, the electromigration of ions under the influence of an electric field, and reaction kinetics. The equation is complex due to the inclusion of terms that account for porosity (ε), tortuosity (τ), the charge number (z^i^), Faraday's constant (F), the universal gas constant (R), temperature (T), and the electric potential (Φ_liq_ and Φ_sol_). This equation, along with others in the system, enables the modeling of ion transport behavior in an electrolytic cell like the Hall-Héroult cell for aluminum production [9,10]:

(43) $$\begin{array}{ccc} & & \int \sum_{i}\delta c_{i}[\varepsilon \frac{\partial c_{i}}{\partial t}+\nabla \cdot \left(-\varepsilon \frac{D_{i}}{\zeta^{2}}\nabla c_{i}-z_{i}c_{i}F\varepsilon \frac{u_{i}}{\zeta^{2}}\nabla \phi_{liq}-\varepsilon c_{i}\nabla \Pi \right)\\ & & +\;\varepsilon z_{i}\lambda +A\frac{\chi_{i}S_{i}}{RT}i_{o}\left(\phi_{sol}-\phi_{liq}-U\right)-\varepsilon k\psi_{i}s_{i}\left[K-\frac{a_{NaF}^{2}c_{AlF_{4}^{-1}}}{c_{AlF_{6}^{-3}}}\right]]\\ & & i=Na^{+},F^{-1}{,\text{AlF}}_{4}^{-1},AlF_{6}^{-3},Al^{0} \end{array}$$

The relationship between Eqs. (42) and (43) lies in their shared function of modeling ionic species conservation within different domains of a Hall-Héroult electrolytic cell. Eq. (42) is specific to the non-porous domain (likely the bulk liquid electrolyte), while Eq. (43) extends this modeling to account for the porous domain (such as a porous cathode structure impregnated with electrolyte). Both equations are structured similarly, signifying that the same fundamental processes such as ionic diffusion, migration under an electric field, and reaction kinetics are at play in both domains. However, Eq. (43) includes additional factors to account for the effects of the porous structure, such as the porosity (ε) and tortuosity (τ), which modify the effective transport properties within the porous medium.

The equations for the potential are those obtained from the conservation of charges. Eqs. (44) and (45) are defined for the liquid phase potential in the non-porous and porous domains. Eq. (46) is defined as the conservation of charges in the solid phase of the porous medium. The following Eqs. (44), (45), and (46) pertain to the potential in the liquid and solid phases within the Hall-Héroult cell. They are derived from the conservation of charges for the non-porous and porous liquid phases and for the solid phase of the porous medium, respectively [9], [10], [11]:

(44) $$\begin{array}{ccc} & & \int \delta \Phi_{liq}\nabla \cdot \left[F\sum_{i}z_{i}\left(-D_{i}\nabla c_{i}-z_{i}c_{i}Fu_{i}\nabla \Phi_{liq}\right)\right]dV=0\\ & & i=Na^{+},F^{-}{,\text{AlF}}_{4}^{-},{\text{AlF}}_{6}^{-3},Al^{0} \end{array}$$

(45) $$\begin{array}{c} \int \delta \Phi_{liq}\left[\nabla \cdot \left[F\sum_{i}z_{i}\left(-\frac{D_{i}}{\zeta^{2}}\varepsilon \nabla c_{i}-z_{i}c_{i}F\frac{u_{i}}{\zeta^{2}}\varepsilon \nabla \Phi_{liq}\right)\right]-A\frac{i_{0}nF}{RT}\left(\Phi_{sol}-\Phi_{liq}-U\right)\right]=0\\ i=Na^{+},F^{-}{,\text{AlF}}_{4}^{-},{\text{AlF}}_{6}^{-3},Al^{0} \end{array}$$

(46) $$\int \delta \Phi_{liq}\left[\nabla \cdot \left[-\left(1-\varepsilon \right)\frac{\kappa_{sol}}{\zeta_{sol}^{2}}\nabla \Phi_{sol}\right]+A\frac{i_{0}nF}{RT}\left(\Phi_{sol}-\Phi_{liq}-U\right)\right]dV=0$$

In relation to the use of the Lagrange multiplier method to impose electroneutrality, Eq. (S27) must also be solved (see supplementary Eq. (S27) and Tables S3 & S4). Thus, the below Eq. (47) associated with this Lagrange multiplier method, is used to enforce electroneutrality within these phases. It's integrated into the model as a constraint equation to ensure that the charge conservation principle is not violated, which is critical in electrochemical systems to avoid the buildup of charge that can lead to unrealistic physical predictions [9,10]:

(47) $$\int \delta \lambda \left(\sum_{i}z_{i}c_{i}\right)dV=0i=Na^{+},F^{-}{,\text{AlF}}_{4}^{-}{,\text{AlF}}_{6}^{-3},Al^{0}$$

The version for the porous medium of Eq. (47) is simply the multiplication of the latter by the porosity. To complete the system, the conservation of mass equations for the liquid phase for the porous and non-porous domain are needed to evaluate the velocity field as shown in Eqs. (48) and (49). Table 4 describes the main parameters used in the numerical simulations with a system maintained at a temperature of 1300 K [17], [18], [19], [20].

(48) $$\int \delta \Pi \left[\frac{\partial \rho }{\partial t}+\nabla \cdot \left(-\rho \nabla \Pi \right)\right]dV=0$$

(49) $$\int \delta \Pi \left[\varepsilon \frac{\partial \rho }{\partial t}+\nabla \cdot \left(-\varepsilon \rho \nabla \Pi \right)\right]dV=0$$

**Table 4:** Basic parameters for the porous electrode (cathode) model.

Parameters	Values						
K	0.04312						
T	1300K						
*i*_0_[18,19]	$12\times 10^{4}A\cdot m^{-2}$ (5 % Al_2_O_3_ and 1283 K)						
A [17]	$17\times 10^{3}m^{2}\times m^{-3}$						
ε	0.2						
N	3						
$\begin{array}{c} U\left(CR\right)=U\left(C_{Na^{+}},C_{AlF_{4}^{-}},C_{AlF_{6}^{-3}}\right) \end{array}$	$U=(\frac{0.6012}{CR-0.4587})-1.011$						
ζ	Eq. (36)						
$\zeta_{sol}$	Eq. (40)						
K	$1\times 10^{10}$						
$\kappa_{sol}$[20]	$2\times 10^{5}\Omega^{-1}\cdot m^{-1}$ (Graphitized cathode, 500 K)						


Species	Di $\left(m^{2}s^{-1}\right)$	u_i_ = D_i_/RT	$z_{i}$	$\chi_{i}$	$S_{i}$	$\psi_{i}$	$s_{i}$
$Na^{+}$	9.43 × 10^−9^	8.7215 × 10^−13^	+1	0	0	0	0
$F^{-}$	4.63 × 10^−9^	4.2818 × 10^−13^	−1	1	4	1	2
${\text{AlF}}_{4}^{-}$	4.05 × 10^−9^	3.76234 × 10^−13^	−1	−1	1	1	1
${\text{AlF}}_{6}^{-3}$	3.12 × 10^−10^	2.8914 × 10^−14^	−3	0	0	−1	1
$Al^{0}$(dissolved) (Thonstad et al. [20])	1.08 × 10^−6^	9.971 × 10^−11^	−1	1	1	0	0

These above equations are essential for calculating the velocity field of the liquid phase within the Hall-Héroult cell. They facilitate the understanding of how the velocity of the liquid phase evolves over time, which is crucial for predicting the behavior of the electrolytic bath in both the non-porous and porous regions of the cathode. The system is maintained at an elevated temperature, characteristic of the operational conditions of the Hall-Héroult process.

**Fig. 4:**
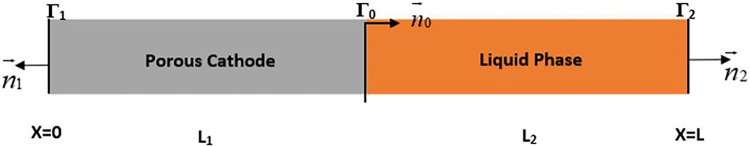
1D Schematic of the conduction system in an aqueous medium with ionic equilibrium (x ∈ [0, L], *L* = 0.045 m, L_1_ = 0.015 m, L_2_ = 0.030 m).

The system is 1D, consisting of a carbon block (the cathode) with a depth of L_1_ = 0.015 m, on which a liquid domain with a depth of L_2_ = 0.030 m rests. The geometry is a straight line shown in Fig. 4. The cathode and the supernatant liquid phase interface are located at the Γ_0_ boundary. The boundary conditions on the fluid's upper face (Γ_2_ boundary) are Dirichlet type for the liquid phase (ci, Ф_liq_). The boundary conditions (Γ_1_ boundary) at the bottom of the cathode are of Neumann type for the solid phase (Ф_sol_) and liquid phase (c_i_, Ф_liq_). Only the solid phase has a boundary condition at the Γ_0_ interface between the cathode and the supernatant bath. The other variables are continuous at the interface.

The system starts at thermodynamic, electrical equilibrium and is electron-to-electron. The composition of the starting liquid phase is CR 1.85 and is imposed as the composition at the Dirichlet-type boundary for the ionic species. The current density imposed at the bottom of the solid is 0.7 cm^−2^. The surface current density of the liquid phase is assumed to be zero (insulating condition) at the bottom of the cathode. The studies of various initial and boundary conditions are presented in Tables 5 and 6. Normal boundary conditions are positive and definite when they point outside the domain. The initial and boundary conditions in Tables 5 and 6 respect the electroneutrality criterion and the ionic balance.

**Table 5:** Initial conditions for the liquid phase at CR = 1.853.

Parameters	Values	Parameters	Values
$C_{Na^{+}}\left(x,0\right)$	2.2577 × 10^4^	$\Phi_{liq}\left(x,0\right)$	−0.589972 V [1]
$C_{F^{-}}\left(x,0\right)$	2.393 × 10^3^	$\Phi_{sol}\left(x,0\right)$	−0.589972 V [1]
$C_{AlF_{4}^{-}}\left(x,0\right)$	9.2123 × 10^3^	λ(x,0)	1
$C_{AlF_{6}^{-3}}\left(x,0\right)$	3.9991 × 10^3^	Π(x,0)	0
$C_{Al^{0}}\left(x,0\right)$	0

**Table 6:** Boundary conditions used in this study.

Boundary Conditions	
$-{\vec{n}}_{1}\cdot {\vec{J}}_{Na^{+}}^{liq}\left(0,t\right)=0$	$C_{Al_{dissolved}^{0}}\left(L,t\right)=C_{Al^{0}}\left(x,0\right)$
$C_{Na^{+}}\left(L,t\right)=C_{Na^{+}}\left(x,0\right)$	$\Phi_{liq}\left(L,t\right)=\Phi_{liq}\left(x,0\right)$
$-{\vec{n}}_{1}\cdot {\vec{J}}_{F^{-}}^{liq}\left(0,t\right)=0$	$-{\vec{n}}_{1}\cdot {\vec{i}}^{liq}\left(0,t\right)=0$
$C_{F^{-}}\left(L,t\right)=C_{F^{-}}\left(x,0\right)$	$-{\vec{n}}_{1}\cdot {\vec{i}}^{liq}\left(0,t\right)=-7000Am^{-2}$
$C_{AlF_{4}^{-}}\left(L,t\right)=C_{AlF_{4}^{-}}\left(x,0\right)$	λ(L,t)=λ(x,0)
$-{\vec{n}}_{1}\cdot {\vec{J}}_{AlF_{6}^{-3}}^{liq}\left(0,t\right)=0$	–
$C_{AlF_{6}^{-3}}\left(L,t\right)=C_{AlF_{6}^{-3}}\left(x,0\right)$	Π(L,t)=0
$-{\vec{n}}_{1}\cdot {\vec{J}}_{Al^{0}}^{liq}\left(0,t\right)=0$	$-{\vec{n}}_{1}\cdot \left[-\nabla \Pi (0,t)\right]=0$

The final section of our study is dedicated to the rigorous numerical validation tests conducted on a half-electrolysis cell, focusing on ion profiles, the concentration profile of dissolved aluminum metal, density, charge ratio (CR), potential values, and system flows. Two additional models were employed for comparative analysis: one without considering charge attribution to dissolved aluminum and another without accounting for convection effects. The section concludes with a meticulous error analysis concerning electroneutrality and ionic equilibrium, revealing results of exceptionally high accuracy. Supplementary Table S6 provides a detailed account of these errors in electroneutrality and ionic equilibrium and their respective variants, all demonstrating remarkable precision.

**Fig. 5:**
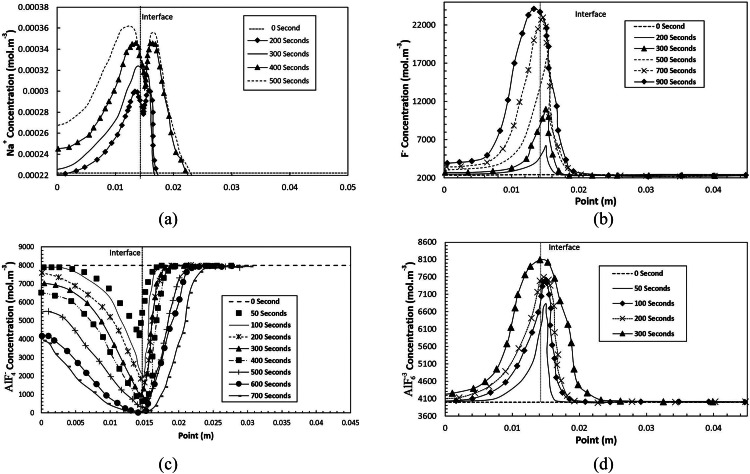
Molar concentration of Na^+^, *F*^−^,${\text{AlF}}_{4}^{-}$,${\text{AlF}}_{6}^{-3}$species as a function of point (m): (a) Na^+^ at time *t* = 0**–**500 s; (b) *F*^−^specie at time *t* = 0**–**900 s; (c) ${\text{AlF}}_{4}^{-}$ specie at time *t* = 0–900 s; (d) ${\text{AlF}}_{6}^{-3}$at time *t* = 0–300 s.

Fig. 5 (a) visually portrays the evolution of Na^+^ ion concentration. Over time, the concentration of Na^+^ ions exhibits an overall increase within the cathode pores. However, as we approach the 200 s mark, a localized decrease in concentration near the cathode-bath interface becomes evident. Additionally, the Na^+^ front extends toward the anode within the free bath zone. In Fig. 5 (b), we observe the evolution of the F- ion concentration, which consistently rises within the pores. Yet, in the free bath zone near the interface, the front extends slightly toward the anode compared to its position within the cathode. Fig. 5 (c) presents the concentration evolution of ion, showing a reduction within the pores and near-zero concentration at the interface region around 650 s. Notably, there is a pronounced systematic decrease in concentration within the solid phase pores. Finally, Fig. 5 (d) describes the evolution of ${\text{AlF}}_{6}^{3}$concentration in the pores away from the interface throughout the simulation. However, near the interface, a more intricate pattern emerges. Initially, concentration growth is observed up to 150 s within the porous interface zone, followed by a subsequent decrease to near-zero levels around 650 s. Concurrently, concentration increases near the interface in the free bath region.

Before delving into the ion distribution profiles, it is imperative to grasp that Na^+^and *F*^−^ ions play pivotal roles in governing transport within the system, given their superior transport properties and molar concentrations exceeding CR 4 (Na^+^ consistently holding the highest concentration when CR >1). It's crucial to bear in mind that ion concentrations within the pores tend to rise at a faster rate than in the free bath region due to the relatively lower pore volume. Our model utilizes a pore volume homogenization method, ensuring the continuity of the liquid phase between the bath contained in the pores and the free bath. However, the transition from the porous to non-porous medium at the pore size scale is not explicitly modeled. At the simulation's outset, the increase in Na^+^ ion concentration within the pores is primarily attributed to their migration under the influence of the electric field, with the cathode being negatively polarized. The limited extension of the Na^+^ front in the free bath zone, in close proximity to the interface, results from diffusion. Negative ions, including F, instigate this diffusive flux^−^, and dissolved aluminum (considered partially charged −1). The diffusive Na^+^ flux contributes significantly to maintaining bath electroneutrality.

The observed trough in Na^+^ ion concentration within the pores, evolving over time, can be attributed to the interplay between migration flows toward the cathode and diffusion flows toward the anode. Furthermore, beyond the presence of *F*^−^, the transport coefficients and the latter concentration are notably higher in this zone, leading to the dominance of ion F- in this area. As a result, it facilitates the rapid transport of Na^+^ ions into adjacent zones. The behavior of ion *F*^−^ is profoundly influenced by the fact that the cathodic electrochemical reaction serves as a substantial source of this ion. Specifically, the consumption of one [Another Ion] ion generates four *F*^−^ions. Similarly, maintaining ionic balance with a low concentration of ions produces two *F*^−^ ions for each dissociation event. Consequently, variations in the concentration of *F*^−^ ions significantly impact the equilibrium, as the activity of NaF is squared in the definition of ionic equilibrium. The high concentration of *F*^−^ substantially influences the behavior of other ions, with substantial diffusion fluxes of *F*^−^ towards both the anode and cathode, augmented by migration flux in the direction of the anode.

The ${\text{AlF}}_{4}^{-}$ ion's behavior remains simpler, and the concentration constantly decreases in the pores because the ion is consumed to produce aluminum. The significant decrease in the concentration of ${\text{AlF}}_{4}^{-}$ ions in the pores suggests that the concentration of ${\text{AlF}}_{6}^{-3}$ ions should also decrease after a significant period of time. The appearance of an overvoltage is associated with the ionic balance between ${\text{AlF}}_{4}^{-}$ and ${\text{AlF}}_{6}^{-3}$ should appear over time in accordance with electrochemical kinetic measurements [24]. The enrichment of the pores in NaF is consistent with what is observed during cathode autopsies. On the other hand, the increase in NaF is probably slower because the formation of metallic sodium slows down the process. The numerical simulation beyond 650 s requires the addition of metallic sodium to the model and possibly an electrochemical kinetic equation, including an exchange current density as a function of the surface concentration of the ion ${\text{AlF}}_{4}^{-}$ and ${\text{AlF}}_{6}^{-3}$.

It should be noted that the concentrations of the ions are similar to those obtained for the straightforward model of Thonstad et al. [18,19]. Near the interface, in the free bath zone and the pores, an increase in the ${\text{AlF}}_{6}^{-3}$ion concentration is caused by the migration towards the anode. Still, this effect attenuates with time as the scattering flux towards the cathode increases in importance. On the other hand, the latter's behavior in the pores is dominated (the increase in concentration) by maintaining the ionic balance indirectly controlled by the *F*^−^ and ${\text{AlF}}_{4}^{-}$concentrations and Na^+^migration flux. Beyond 300 s, the ion profiles hide a concentration stack effect that is not very apparent without analyzing the voltage curves and the detail of the ion currents and fluxes. This aspect is discussed later in this section after presenting the potential curves.

**Fig. 6:**
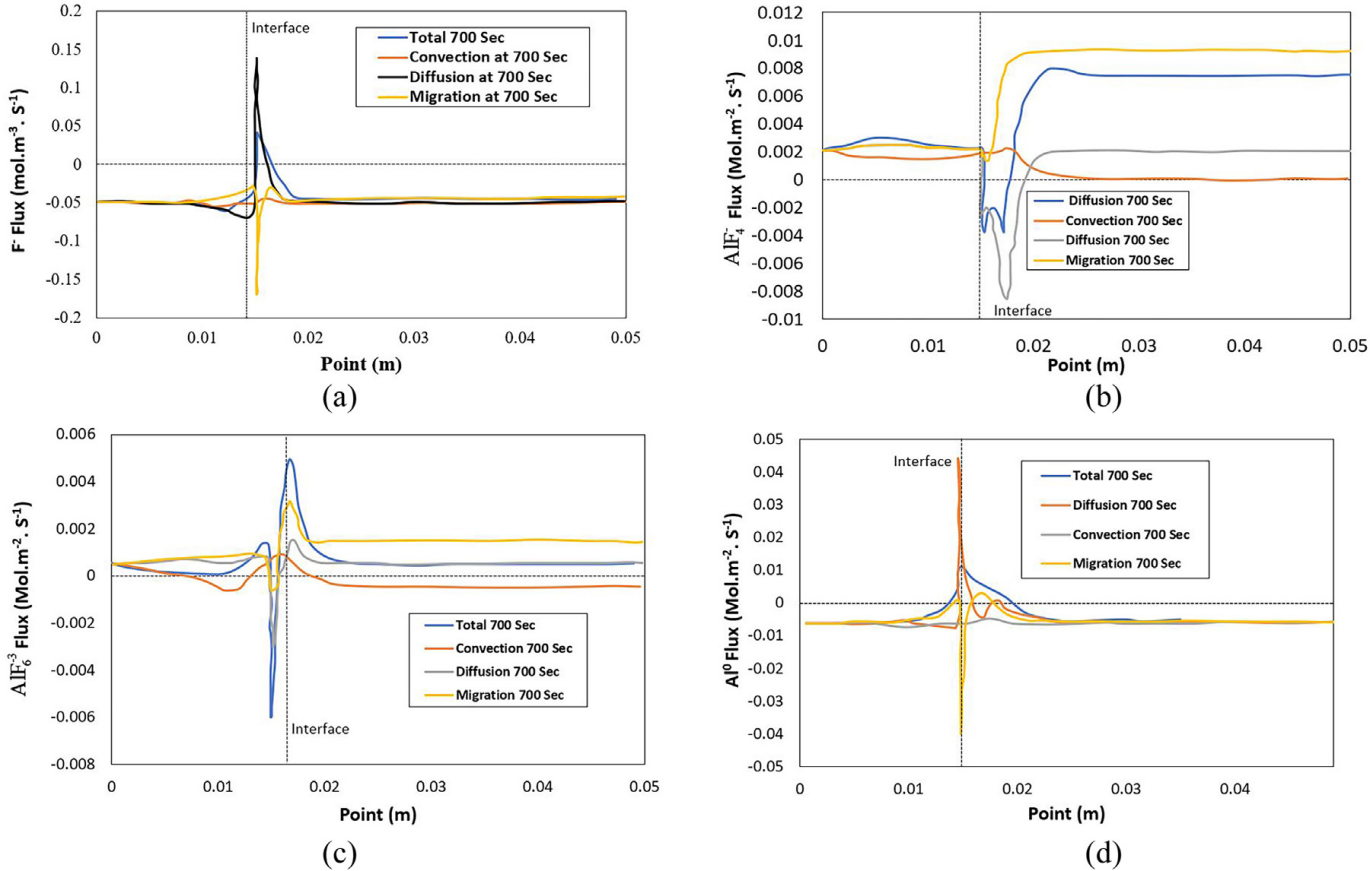
Superficial molar flux of $F^{-}$, ${\text{AlF}}_{4}^{-}$, ${\text{AlF}}_{6}^{-3}$,$Al^{0}$ as a function of the point at 700 s : (a) $F^{-}$; (b) ${\text{AlF}}_{4}^{-}$; (c) ${\text{AlF}}_{6}^{-3}$; (d) $Al^{0}$.

Fig. 6 (a) shows that the F- net molar flux is in the anode direction and is dominated by the diffusion in the free bath zone and the migration in the cathode pore. A weak net flux towards the cathode is present far from the interface. This flux contributes to the CR increase at the depth of the cathode. Fig. 6 (b) and (c) show that the net flux of ${\text{AlF}}_{4}^{-}$ and ${\text{AlF}}_{6}^{-3}$ ions are oriented towards the anode except in the concentration pile area and the porous medium ${\text{AlF}}_{6}^{-3}$. The diffusion and migration fluxes for ${\text{AlF}}_{4}^{-}$ and ${\text{AlF}}_{6}^{-3}$ ions generally progress in the same direction, contrary to the other ions. In other words, the migration varies in phase with the diffusion in the same direction except in the concentration stack zone. The convection and migration fluxes are dominant for the ${\text{AlF}}_{4}^{-}$ and ${\text{AlF}}_{6}^{-3}$ions, while the migration and diffusion fluxes are dominant for the Na^+^ and *F*^−^ions. Fig. 6 (d) shows the net flux of dissolved aluminum, primarily oriented towards the anode and spreading more evenly than other ionic species, with a behavior identical to the *F*^−^ ion. Fig. 6(a) – (d) show that the reverse migration zone linked to the concentration pile is not dominant, and the diffusivity controls the current in this zone, which does not lead to a direction change of the latter. The concentration profiles of ions within the electrolysis cell offer insights into the dynamic interplay of transport mechanisms. As Na^+^ ions migrate under the influence of the electric field and current density, their gradual increase in concentration within the pores is observed, particularly during the initial stages of the simulation. However, this increase is counteracted by the diffusion flow toward the anode. As time progresses, an intriguing phenomenon emerges where a decrease in local Na^+^ concentration occurs near the cathode-bath interface. This decrease is attributed to the dominance of *F*^−^ions, which exhibit higher transport coefficients and concentrations. *F*^−^ ions, generated by the cathodic electrochemical reaction, play a pivotal role in maintaining ionic balance. The diffusion flux of *F*^−^ towards both the anode and cathode, coupled with migration, contributes significantly to ion transport dynamics.

Moreover, the concentration profiles reveal the formation of a concentration pile, a phenomenon not readily apparent without a detailed analysis of voltage curves and ion currents. These intricate ion behaviors highlight the significance of maintaining electroneutrality and ionic equilibrium within the system. The concentrations of Na^+^ and *F*^−^ ions, as the dominant transport species, substantially impact the behavior of other ions. The diffusion and migration fluxes of Na^+^ and *F*^−^ions exhibit distinct characteristics, influencing the direction and rate of ion transport.

On the other hand, Al^3+^ ions, integral to aluminum production, exhibit a unique behavior as they are consumed in the process, leading to a gradual decrease in their concentration within the pores. This decline in Al^3+^ concentration aligns with electrochemical kinetic measurements, indicating the emergence of an overvoltage associated with ionic balance. Further model refinements may involve the incorporation of metallic sodium and electrochemical kinetic equations to capture these complex dynamics beyond the simulation's current timeframe.

### Conclusion

In this seminal study, we embark on a pioneering journey to unravel the complex dynamics governing the electrochemical degradation of the porous electrode within the Hall-Héroult cell cathode. Our endeavor is underpinned by the development of an advanced model that sheds light on these intricate processes and introduces a groundbreaking numerical method. The primary objective is maintaining electroneutrality and upholding ionic thermodynamic equilibrium with unprecedented precision.

We dissect the multifaceted world of cathode degradation with meticulous scrutiny, leading us to craft a detailed and comprehensive porous electrode model. This model serves as the linchpin of our research, harnessing a sophisticated homogenization method that faithfully replicates the transport and interactions of multiple ionic species.

A hallmark of our study is the innovative application of the Lagrange multiplier within the finite element analysis framework. This ingenious approach enhances our model's fidelity to electroneutrality and sets a new standard for numerical techniques in this domain.

As we navigate through our findings, we take pride in showcasing the model's alignment with empirical data, a testament to its accuracy and reliability. However, we acknowledge that the pursuit of scientific excellence is an ongoing endeavor. Therefore, we identify areas for potential refinement, especially in the realm of simulating aluminum dissolution and comprehending the intricate effects of metallic sodium within the system.

In essence, this study not only advances our understanding of cathode degradation in Hall-Héroult cells but also paves the way for future research and innovation in the field of aluminum production. Our work is a testament to the relentless pursuit of knowledge and the unwavering commitment to excellence in scientific inquiry.

### Supplementary material and/or additional information [OPTIONAL]

The supplementary material file contains the following components:

1. Electroneutrality by Elimination Method:
  • Describes a classical approach to achieve electroneutrality by eliminating the conservation equation of a cation or anion.
  • Provides mathematical equations and steps for eliminating species.
  • Discusses the use of the Nernst-Planck application mode for finite element resolution.
2. Electroneutrality by Constraint:
  • Introduces an alternative method to maintain electroneutrality without eliminating species equations.
  • Explains the application of a constraint through variational forms.
  • Discusses the formulation for minimizing the solution with respect to the constraint.
3. Ionic Equilibrium Implementation:
  • Discusses the approximation of ionic equilibrium in a transient species conservation problem.
  • Analyzes three methods for implementing ion balance.
  • Mentions the importance of prioritizing the resolution of partial differential equations.
4. Evaluation of Ionic Equilibrium Methods:
  • Presents a case study involving a conductivity problem in an aqueous medium with ion balance.
  • Compares various methods for implementing ionic equilibrium and electroneutrality.
  • Describes the parameters used for numerical resolution with finite element methods (FEM).
5. Figures and Tables:
  • Shows concentration results for different ions and potential in the case study.
  • Summarizes the performance of the methods in terms of electroneutrality and ionic balance.

The document evaluates different methods for achieving electroneutrality and ionic balance in a complex system and compares their effectiveness in a case study involving ion conduction in an aqueous solution. The results indicate that various methods may yield similar results but should be evaluated based on multiple criteria beyond electroneutrality.

### Ethics statements

Our work presented in this MethodsX article did not involve human subjects, animal experiments, or the collection of data from social media platforms. Therefore, the relevant informed consent, ethical guidelines for animal experiments, and compliance with social media data policies are not applicable to this research. This work strictly adheres to ethical standards and guidelines relevant to numerical simulations and computational modeling.

### CRediT authorship contribution statement

**Yun Peng Zhang:** Conceptualization, Investigation, Visualization, Writing – original draft, Writing – review & editing. **Nan Zou:** Conceptualization, Methodology, Investigation, Writing – original draft, Writing – review & editing. **Shuang Jun Ma:** Methodology, Investigation, Visualization, Writing – review & editing. **Yang Youjian:** Writing – review & editing, Funding acquisition, Supervision. **Mouhamadou A. Diop:** Conceptualization, Methodology, Funding acquisition, Project administration, Supervision, Writing – review & editing.

## Declaration of competing interest

The authors declare that they have no known competing financial interests or personal relationships that could have appeared to influence the work reported in this paper.

## Data Availability

Data will be made available on request.

## Appendix Supplementary materials

Image, application 1

Image, application 2
