# Supplementary material for: Advancing the prediction of bath penetration and electrochemical degradation in Hall-Héroult cell cathodes: Insights into ionic species transport in a porous electrode model
Source: MethodsX. 2024 Feb 5;12:102593. doi: 10.1016/j.mex.2024.102593 (PMC11636911; doi:10.1016/j.mex.2024.102593)
Supplement: Supplementary file 1 [file mmc1.pdf]

Electronic Supplementary Materials File

Advancing the Prediction of Bath Penetration and Electrochemical Degradation in Hall-Héroult Cell Cathodes: Insights into Ionic Species Transport in a Porous Electrode Model

Yun Peng Zhang[a], Zou Nan[a], Long Wang[a], ShuangJun Ma[a], Mouhamadou A. Diop[a,b†]

[a]School of Metallurgy, Northeastern University, Shenyang, 110819, P.R. China.

[b]Key Laboratory for Ecological Metallurgy of Multimetallic Minerals (Ministry of Education), Shenyang, 110819, P.R. China.

[†]**Corresponding author:** diop@smm.neu.edu.cn; Tel/Fax: +86 24 8368 6381

Tables:

Table S1. Parameters of study case of conduction in aqueous medium with ionic equilibrium.

**Diffusion coefficients and species' parameters**							
i	z_i	s_i	Ψ_i	D_i			
Na^+	+1	0	0	$1.33x10^{-9} m^2 s^{-1}$			
Cl^-	-1	0	0	$2.03x10^{-9} m^2 s^{-1}$			
Fe^{+2}	+2	1	-1	$0.719x10^{-9} m^2 s^{-1}$			
FeY^{-2}	-2	1	1	$0.4x10^{-9} m^2 s^{-1}$			
Y^{-4}	-4	1	-1	$0.5x10^{-9} m^2 s^{-1}$			
Composition of aqueous solution before application of initial ionic equilibrium and other parameters							
C_{Nacl}	C_{FeCl_2}	C_{Na_4F}	T	K	k	i_0	n
10^{-2}mol m^{-3}	10^{-3}mol m^{-3}	10^{-3}mol m^{-3}	298.15 K	100	$1x10^{12}$	0.02 $A.m^{-2}$	2

Table S2. Initial and boundary conditions of condition problem in aqueous medium with ionic equilibrium (x $\in$ [0, L], L = 0.001m).

**Boundary Conditions**	
$C_{Na^+}(x,0)$	$0.014\ molm^{-3}$
$C_{Cl^-}(x,0)$	$0.012\ molm^{-3}$
$C_{Fe^{+2}}(x,0)$	$9.160798\times10^{-4}\ molm^{-3}$
$C_{Y^{-4}}(x,0)$	$9.160798\times10^{-4}\ molm^{-3}$
$C_{FeY^{-2}}(x,0)$	$8.392022\times10^{-5}\ molm^{-3}$
$\Phi(x,0)$	0V
$\lambda(x,0)$	1

**Boundary Conditions**	
Γ_1	Γ_2
$-\vec{n}_1\cdot\vec{J}_{Na^+}=0\ mol\cdot m^{-2}\cdot s^{-1}$	$C_{Na+}(L,t)=0.014\ mol\cdot m^{-3}$
$-\vec{n}_1\cdot\vec{J}_{Cl^-}=0\ mol\cdot m^{-2}\cdot s^{-1}$	$C_{Cl-}(L,t)=0.012\ mol\cdot m^{-3}$
$-\vec{n}_1\cdot\vec{J}_{Fe^{2+}}=\frac{i_0}{nF}$	$C_{Fe^{2+}}(L,t)=9.160798\times10^{-4}\ mol\cdot m^{-3}$
$-\vec{n}\cdot\vec{J}_{Y^{-4}}=0\ mol\cdot m^{-2}\cdot s^{-1}$	$C_{Y^{-4}}(L,t)=9.160798\times10^{-4}\ mol\cdot m^{-3}$
$-\vec{n}\cdot\vec{J}_{FeY^{-2}}=0\ mol\cdot m^{-2}s^{-1}$	$C_{FeY^{-2}}(L,t)=8.392022\times10^{-5}\ mol\cdot m^{-3}$
$-\vec{n}\times\vec{i}=0.02\ A\times m^{-2}$	$\Phi(L,t)=0\text{V}$
	$\lambda(L,t)=1$

Table S3. Methods used in this study case.

**Methods**	Ionic Equilibrium			Electroneutrality	
	Elimination	Source Term	Penalization	Elimination	Lagrange Multiplier
PenElim			X	X	
PenMulti			X		X

Table S4. Resolution parameters used in COMSOL Multiphysics 6.0 for the numerical simulations with FEM.

Parameters	Values
Consistent initialization of PDE-algebraic system	Backward Euler
Implicit resolution method with variable order and variable time step	BDF (Backward differentiation formula)
Time step	$t \in [0, 100\text{s}]$
Direct linear solver	UMFPACK
Constraints treatment method	Elimination
Relative tolerance	10^{-3}
Absolute tolerance	10^{-4}
Meshing in Comsol 6.0 Multiphysics	Free mesh
Element growth rate	1.4
Maximum element size	1x10-6 m
Maximum element dimension scale factor	1
Application mode	General form of PDE
Methods	DOF
PenElim	5015
PenMulti	7037

Table S5. Verification of electroneutrality on the resolution domain at 60 seconds.

**Methods**	**Electroneutrality** $\sum_i z_i c_i,\ i = \text{Na}^+, Cl^-$
Elimination method	0
Lagrange type of constraint	[$-5.6\text{x}10^{-17}$,$4.2\text{x}10^{-17}$]
Nernst-Planck in COMSOL 6.0 Multiphysics	0

Table S6. Verifying electroneutrality and respect of ionic balance in the 100 s resolution domain.

**Methods**	**Relative error on ionic equilibrium:** $100\left[K - \frac{C_{FeY^{-2}}}{C_{Fe^{+2}}C_{Y^{-4}}}\right]\Big/K$	**Electroneutrality:** $\sum_{i=1}^{5} z_i c_i$
PenElim	$[0, 0.187\%]$	$\pm 5.24\times10^{-18}$
PenMulti	$\pm 1\times10^{-3}\%$	$\pm 2.4\times10^{-17}$

Table S7. Errors in different models used.

**Models**	**Relative error on ionic equilibrium** $\frac{100(K-K_{calculated})}{K}$	**Electroneutrality** $\sum_i c_i z_i$
Classical method	[-0.006%,0.066%]	$\pm 2\text{x}10^{-11}$
Without convection	±0.14%	$\pm 2\text{x}10^{-11}$
With $z_{Al^0} = 0$	±0.19%	$\pm 2\text{x}10^{-11}$

<u>Figures:</u>

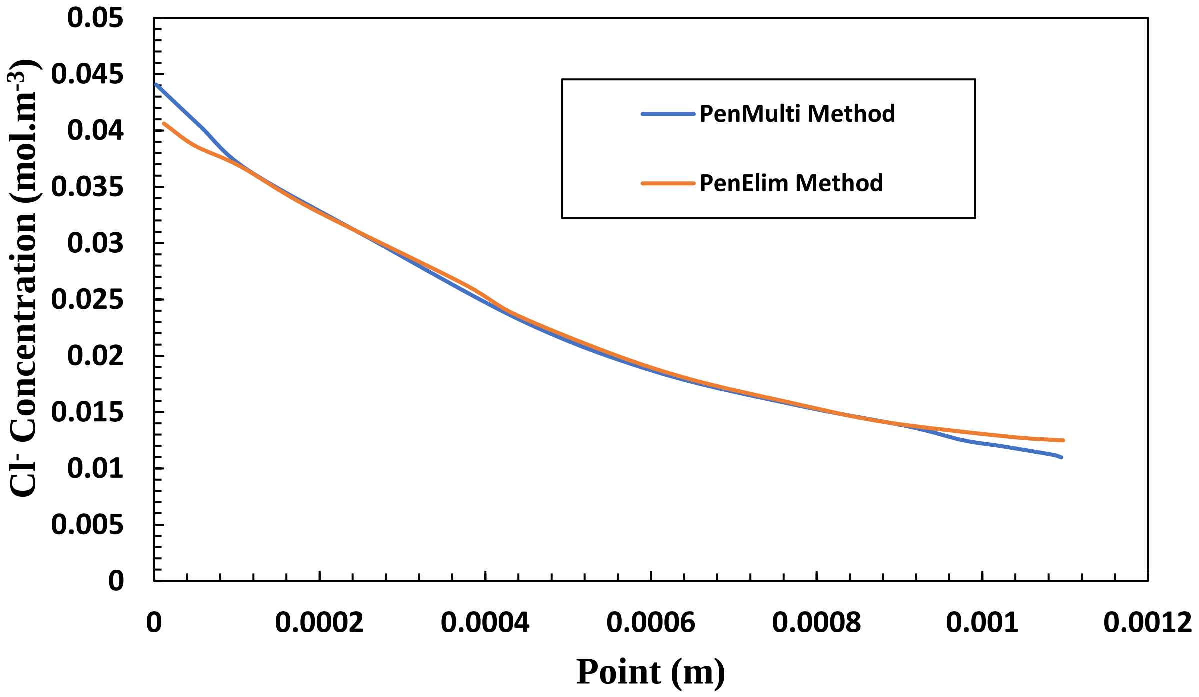

(a)

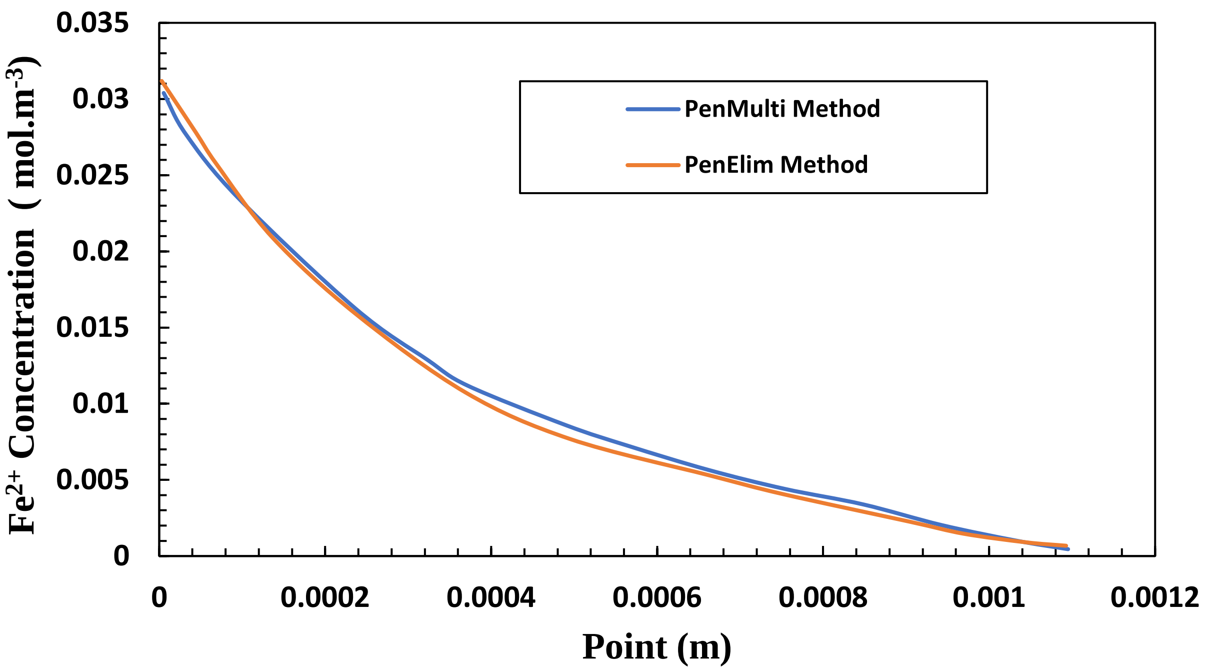

(b)

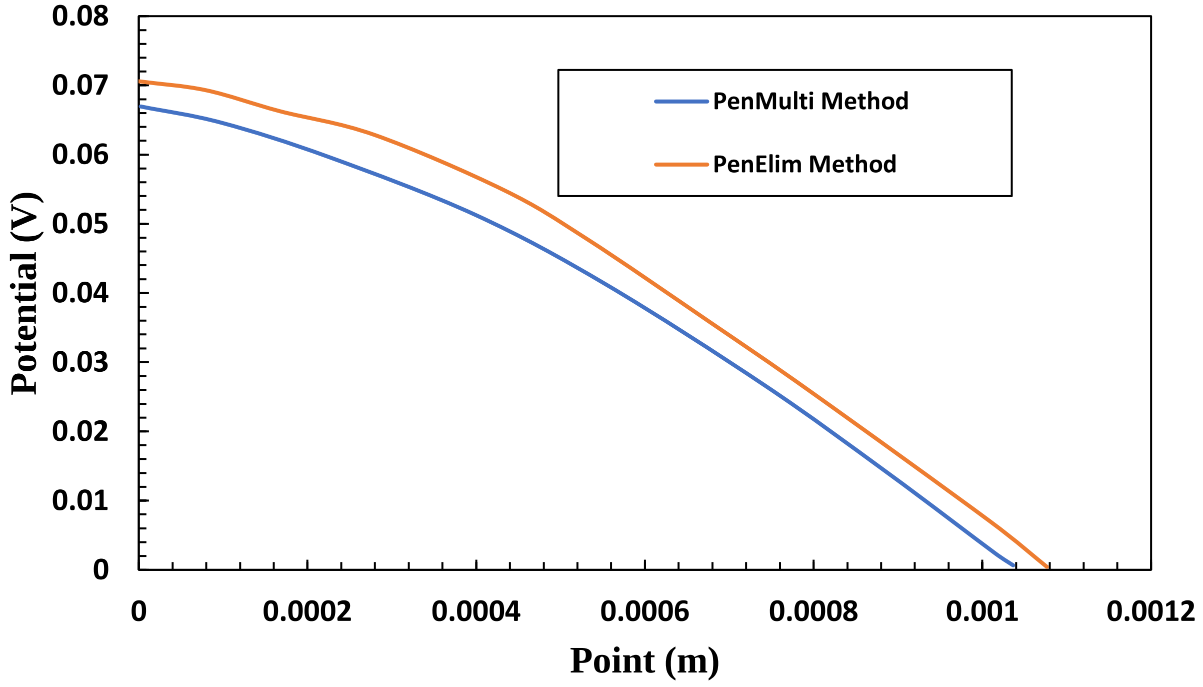

(c)

Fig.S1: Evolution of species concentration in the aqueous solution as the function of the point at time 100 seconds calculated through PenElim and PenMulti methods: (a) Cl^- species; (b) Fe^{2+} and (c) the potential.

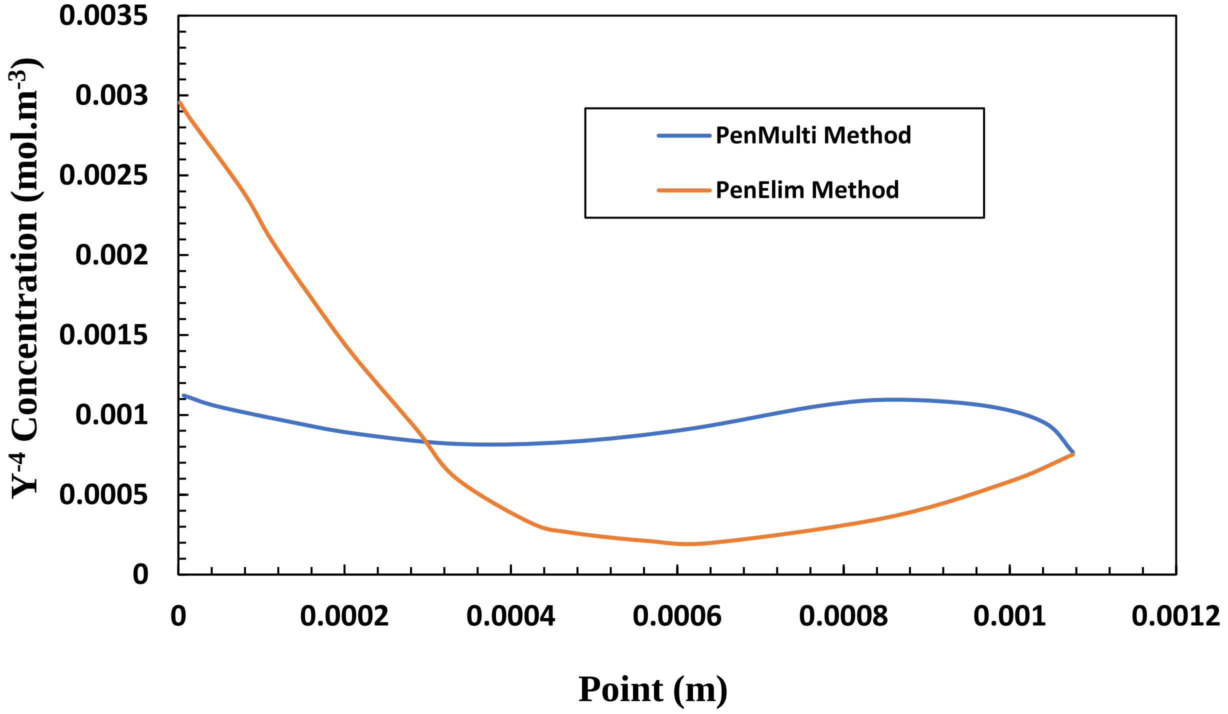
0.0035
0.003
0.0025
0.002
0.0015
0.001
0.0005
0
Y^{-4} Concentration (mol.m^{-3})
PenMulti Method
PenElim Method
0
0.0002
0.0004
0.0006
0.0008
0.001
0.0012
Point (m)

(a)

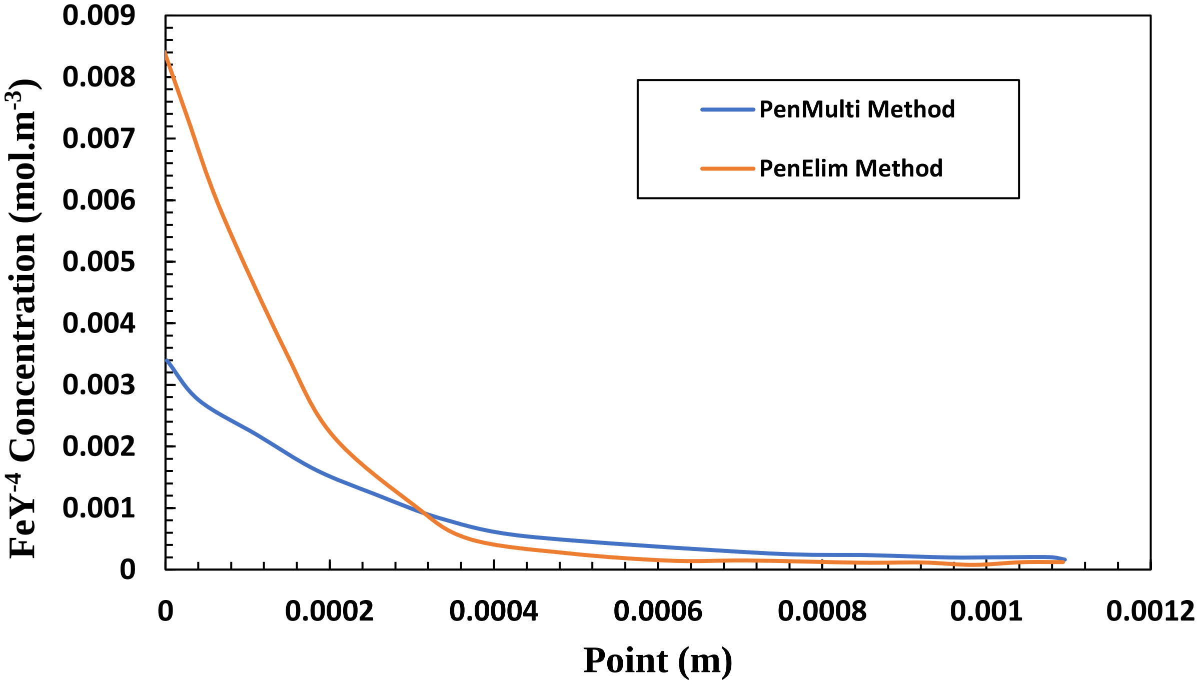
0.009
0.008
0.007
0.006
0.005
0.004
0.003
0.002
0.001
0
FeY^{-4} Concentration (mol.m^{-3})
PenMulti Method
PenElim Method
0
0.0002
0.0004
0.0006
0.0008
0.001
0.0012
Point (m)

(b)

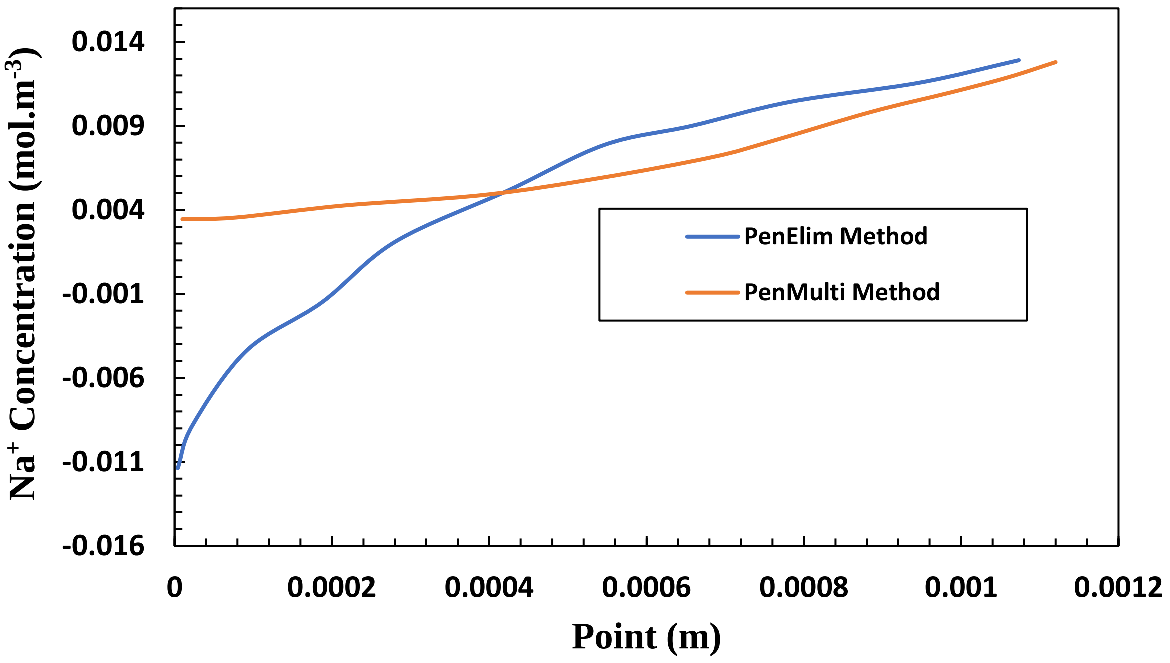

(c)

Fig. S2. Evolution of species concentration in the aqueous solution with ionic equilibrium as a function of the point at time t =100 seconds calculated through PenElim and PenMulti methods: (a) species $Y^{-4;}$ (b) species FeY^{-2}; (c) Na+ specie.
