# Supplementary material for: Advancing the prediction of bath penetration and electrochemical degradation in Hall-Héroult cell cathodes: Insights into ionic species transport in a porous electrode model
Source: MethodsX. 2024 Feb 5;12:102593. doi: 10.1016/j.mex.2024.102593 (PMC11636911; doi:10.1016/j.mex.2024.102593)
Supplement: Supplementary file 2 [file mmc2.pdf]

Supplementary Materials

Advancing the Prediction of Bath Penetration and Electrochemical Degradation in Hall-Héroult Cell Cathodes: Insights into Ionic Species Transport in a Porous Electrode Model

Long Wang[a], Zou Nan[a], Yun Peng Zhang[a], Shuangjun Ma[a], Mouhamadou A. Diop[a,b†]

[a]School of Metallurgy, Northeastern University, Shenyang, 110819, P.R. China.

[b]Key Laboratory for Ecological Metallurgy of Multimetallic Minerals (Ministry of Education), Shenyang, 110819, P.R. China.

[†]**Corresponding author:** diop@smm.neu.edu.cn; Tel/Fax: +86 24 8368 6381

This PDF file includes the following:

Materials and Methods

Figs. S1 to S2

Tables S1 to S5

References

1. Electroneutrality by Elimination Method

The classical method to ensure compliance with electroneutrality is to eliminate the conservation equation of a cation or anion. The electroneutrality equation obtains the concentration for the anion or cation whose conservation equation has been eliminated. For example, suppose a system of N species has N conservation equations and the charge conservation equation. The system of equations is defined from Eqs. (4), (29), (S.1), and (S.2) are presented in weighted form,

$$\int \sum_{i=1}^{N} \delta c_i \left[\frac{\partial c_i}{\partial t} + \nabla \cdot \left(-D_i \nabla c_i - z_i c_i F u_i \nabla \phi_{liq} \right) - R_i \right] dV = 0 \qquad \text{(S.1)}$$

$$\int \sum_{i=1}^{N} \delta \phi_{\text{liq}} \nabla \cdot \left[F \sum_{i=1} z_i \left(-D_i \nabla c_i - z_i c_i F u_i \nabla \phi_{liq} \right) \right] dV = 0 \qquad \text{(S.2)}$$

Let's assume we want to eliminate species l, for example. In that case, the elimination procedure consists in using the electroneutrality equation to eliminate the concentration of species 1 while deleting the conservation equation of species 1. The system of equations to be solved numerically is written as follows:

$$\int \sum_{i=2}^{N} \delta c_i \left[\frac{\partial c_i}{\partial t} + \nabla \cdot \left(-D_i \nabla c_i - z_i c_i F u_i \nabla \phi_{liq} \right) - R_i \right] dV = 0 \qquad \text{(S.3)}$$

$$\int \sum_{i=1}^{N} \delta \phi_{\text{liq}} \nabla \cdot \left[F \left(D_1 \left(\sum_{i=2}^{N} z_i \nabla c_i \right) + F z_1 u_1 \left(\sum_{i=2}^{N} z_i c_i \right) \nabla \phi_{liq} \right) + F \sum_{i=2}^{N} z_i \left(-D_i \nabla c_i - z_i c_i F u_i \nabla \phi_{liq} \right) \right] dV = 0$$

(S.4)

Therefore, the system of equations to be solved numerically is of N equations and N variables. After numerical resolution, the concentration of species l is evaluated with the electroneutrality equation.

$$\mathrm{c}_1 = \frac{-1}{z_1} \sum_{i=2}^{N} z_i c_i \qquad \text{(S.5)}$$

The Nernst-Planck application mode of the finite element resolution software COMSOL Multiphysics 6.0 proceeds by a similar method.

The system of equations for the single elimination method (Eqs. (33), (34), and (35)) is applied to the problem defined in this section. The system to be solved by the finite element method consists of the following two equations:

$$\int \delta \mathrm{c}_{Na^+} \left[\frac{\partial c_{Na^+}}{\partial t} + \nabla \cdot \left(-D_{Na^+} \nabla c_{Na^+} - Z_{Na^+} c_{Na^+} F u_{Na^+} \nabla \phi_{liq} \right) \right] dV = 0 \qquad \text{(S.6)}$$

$$\int \delta \phi_{\text{liq}} \nabla \cdot \left[z_{Na^+} F \left(D_{cl^-} - D_{Na^+} \right) \nabla c_{Na^+} + F^2 \left(z_{cl^-} z_{Na^+} u_{cl^-} - z_{Na^+}^2 u_{Na^+} \right) c_{Na^+} \nabla \phi_{liq} \right] dV = 0 \quad \text{(S.7)}$$

The Nernst-Planck application mode of COMSOL Multiphysics 6.0 is used as a complement to demonstrate the validity of the Lagrange multiplier method. The system of equations for the method with Lagrange constraint is applied to the problem defined in this section. The system to be solved by the finite element method consists of the following four equations:

$$\int \sum_{i} \delta c_i \left[\frac{\partial c_i}{\partial t} + \nabla \cdot \left(-D_i \nabla c_i - z_i c_i F u_i \nabla \phi_{liq} \right) + \lambda z_i \right] dV = 0 \quad i = Na^+, Cl^- \qquad \text{(S.8)}$$

$$\int \delta \phi_{\text{liq}} \nabla \cdot \left[F \sum_{i} z_i \left(-D_i \nabla c_i - z_i c_i F u_i \nabla \phi_{liq} \right) \right] dV = 0 \quad i = Na^+, Cl^- \qquad \text{(S.9)}$$

$$\int \delta\lambda \left(\sum_i z_i c_i \right) dV = 0 \quad i = Na^+, Cl^- \tag{S.10}$$

2. Electroneutrality by Constraint

The choice to use a constraint to apply the electroneutrality criterion helps to avoid eliminating the equation of a species. We can therefore apply boundary conditions to all the species in the system. Moreover, the electroneutrality criterion is a macroscopic constraint not existing at the molecular scale or of the interfacial double layer present during an electrochemical reaction between two phases. This electroneutrality equation is not fundamental [1, 2]. A constraint can be applied to a system of partial differential equations solved by the finite element method by formulating the problem through a variational form. For example, in the case of a species conservation problem involving some constraint depending on the concentration of one or more species c_i, the problem is defined as follows:

$$\delta\Pi^* \left(c_i, \lambda_j \right) = \delta\Pi \left(c_i \right) + \sum_j \delta \int \lambda_j \left[f_j(c_i) \right] dV \tag{S.11}$$

The previous formulation does not guarantee that the functional $\Pi(c_i)$, coming from the partial differential equations describing species conservation, exists. Still, it is nevertheless possible to pose the problem to be able to derive a form minimizing the solution related to the constraint.

The terms coming from the first variation are then directly added to the weighted form $\delta\Pi(c_i)$, which is well-defined according to the weak form of the finite element problem. In the context of this study, the constraint applies to the electroneutrality equation, and we, therefore, have the following functional,

$$\Pi^* \left(ci, \phi_{liq}, \phi_{sol}, \lambda \right) = \delta\Pi \left(ci, \phi_{liq}, \phi_{sol} \right) + \int \delta\lambda \left(\sum_i c_i z_i \right) dV = 0 \tag{S.12}$$

One takes the first variation expression, which is as follows,

$$\delta\Pi^* \left(c_i, \phi_{liq}, \phi_{sol}, \lambda \right) = \delta\Pi \left(c_i, \phi_{liq}, \phi_{sol} \right) + \int \delta\lambda \left(\sum_i c_i z_i \right) dV + \sum_i \int \lambda z_i \delta c_i dV = 0 \tag{S.13}$$

Therefore, the additional equations from the constraint are added to the system to be solved through the weak form. On the other hand, applying a constraint of the Lagrange multiplier type requires adding an additional equation for electroneutrality and one more variable to be solved on the integration domain. Moreover, since we do not eliminate the conservation equation of a species, the system of equations must be solved for this variable. If we use the same system of equations as sub-section 2.3.1, i.e., Eqs. (S.1) and (32), one obtains the following equations from Eqs. (4), (29) -(S.2),

3. Ionic Equilibrium Implementation

The implementation of ionic equilibrium in a transitory species conservation problem is an approximation, and three methods of implanting an ion balance are analyzed in this work. Other works discuss several alternative methods [3-5] but do not offer significant new elements compared to the methods presented in this study. The system being solved has seven unknowns, which requires seven equations. Two of these equations are the electroneutrality and ion equilibrium equations, while the remaining equations are based on the principles of conservation of mass. It is crucial to prioritize the resolution of the partial

differential equations (PDEs) to reflect the fundamental behavior of the problem and to use the other equations as constraints. However, it is possible to eliminate one of the species conservation equations in the case of an ionic equilibrium system involving three ions. This approach is not ideal as it does not consider the eliminated species' transport parameters and does not preserve the conservation of charges. Alternative methods that keep transport parameters should be preferred.

If the kinetic equation related to an ionic balance is known, using the methods discussed in the sub-sections cited above is unnecessary. In the framework of the porous electrode transient model presented in this study, the only ion that can be eliminated for the application of ion balance is the AlF_6^{-3} ion because it is the only one present in the ionic equilibrium not involved in an equilibrium reaction. Within the framework of modeling a Hall-Héroult cell's system, the impossibility of applying boundary conditions on the AlF_6^{-3} ion can be problematic. For example, modeling the formation of solid cryolite Na_3AlF_6 by solidification in cathode pores may require formulating an explicitly defined boundary condition as a function of the AlF_6^{-3} ion flux to be able to describe a liquid bath and solid cryolite interface adequately. Only a transient electrochemical type system with a non-zero net current will be studied in this present study. This is justified by the fact that we are interested in modeling an electrolysis system. Since the typical problem is for dilute and stagnant solutions, convection is neglected.

The kinetic parameters are unknown in the ionic balance case investigated in this study. They are difficult to measure, hence the need to use a formulation based on the equilibrium thermodynamics known for the associated neutral system (Na_3AlF_6). Ion equilibrium is assumed to be fast enough to use an approach based on classical thermodynamics. In the context of the porous electrode transient model presented in this study, the only ion that can be eliminated for the ion equilibrium application is the AlF_6^{-3} ion. This is because it is the only one in the ion equilibrium not involved in an equilibrium reaction or requiring a particular boundary condition. In the context of modeling the system of a Hall-Héroult vessel, the impossibility of applying boundary conditions to the AlF_6^{-3} ion can be problematic. For example, modeling the formation of solid Na_3AlF_6 cryolite by solidification in cathodic pores may require formulating an explicitly defined boundary condition based on the flux of the AlF_6^{-3} ion to be able to describe a liquid bath interface and solid cryolite adequately.

The first and simplest method to implement an ionic equilibrium is to eliminate the conservation equation of one of the species involved in the ion balance but retain the effect of its concentration and its gradient in the conservation equation of the species. The concentration for the ionic species, whose conservation equation has been eliminated, is obtained by the thermodynamic ion equilibrium equation. For example, assume a system of N species with N conservation equations along with the charge conservation equation. If one defines an unspecified ionic equilibrium of equilibrium constant K, the equilibrium equation is written,

$$\ln K = \ln \prod_{i=1}^{n} a_i^{s_i} + \ln \prod_{i=n+1}^{m} a_i^{-s_i} \tag{S.14}$$

The parameters $a_{i,}$ and s_i are respectively the activity and the stoichiometry of the ionic species i for the ionic balance defined by Eq. (S.14). The set of species n is that of the products. The set of species m is that of the reactants defined according to the sense of ion balance (Eq. (S.14)). It should be noted that the N species are not necessarily all involved in the ionic balance. The species not involved in the equilibrium are N-m in number. The activity, a_i, must be able to be explicitly defined according to the concentration of the associated ion c_i.The system is defined from Eqs.(4),(29)-(S1) and presented in weighted forms by FEM such as,

$$\int \sum_{i=1}^{N} \delta c_i \left[\frac{\partial c_i}{\partial t} + \nabla \cdot \left(-D_i \nabla c_i - z_i c_i F u_i \nabla \phi_{liq} \right) - R_i \right] dV = 0 \quad \text{(S.15)}$$

$$\int \delta \phi_{liq} \nabla \cdot \left[F \sum_{i=1}^{N} z_i \left(-D_i \nabla c_i - z_i c_i F u_i \nabla \phi_{liq} \right) \right] dV = 0 \quad \text{(S.16)}$$

by suppressing the specie 1, the elimination procedure consists of using the equilibrium equation to eliminate the conservation equation of species 1. The system of equations to be solved numerically becomes as follows,

$$\int \sum_{i=2}^{N} \delta c_i \left[\frac{\partial c_i}{\partial t} + \nabla \cdot \left(-D_i \nabla c_i - z_i c_i F u_i \nabla \phi_{liq} \right) - R_i \right] dV = 0 \quad \text{(S.17)}$$

$$\int \delta \phi_{liq} \nabla \cdot \left[F \sum_{i=1}^{N} z_i \left(-D_i \nabla c_i - z_i c_i F u_i \nabla \phi_{liq} \right) \right] dV = 0 \quad \text{(S.18)}$$

$$\ln K = \ln \prod_{i=1}^{n} a_i^{s_i} + \ln \prod_{i=n+1}^{m} a_i^{-s_i} \quad \text{(S.19)}$$

The aforementioned Eqs. (S.17) to (S.18) can be solved simultaneously or with a method that proceeds by iterating between the partial differential equations and the ion equilibrium equation.

The second method used retains the conservation equation, which has been eliminated. The method is based on the substitution of a transport equation of a species associated with the equilibrium equation through a source term $\Psi s_i R$ [3-7]. Once we take the same system (Eqs. (S.15) and (S.16)) by introducing a source term for the ionic reaction described by the equilibrium equation (Eq. (S.14)), one obtains the following system,

$$\int \sum_{i=1}^{N} \delta c_i \left[\frac{\partial c_i}{\partial t} + \nabla \cdot \left(-D_i \nabla c_i - z_i c_i F u_i \nabla \phi_{liq} \right) - R_i - \psi_i s_i \Re \right] dV = 0 \quad \text{(S.20)}$$

$$\int \delta \phi_{liq} \nabla \cdot \left[F \sum_{i=1}^{N} z_i \left(-D_i \nabla c_i - z_i c_i F u_i \nabla \phi_{liq} \right) \right] dV = 0 \quad \text{(S.21)}$$

The Ψi parameter has a value of -1 for reactants and +1 for products. The equilibrium implementation method consists in substituting the equation of the species whose equation we want to eliminate (the non-independent species in the thermodynamic sense) by means of the source term. The disadvantage of proceeding with the procedure of elimination by substitution presented in this section is the impossibility of applying boundary conditions of the Neumann type on the variable c_i because its conservation equation is treated as a source term in the other equations. On the other hand, contrary to the elimination method, the transport parameters associated with the species l are present throughout the system of equations.

The last method presented in this section is based on a formulation by a penalization method similar to the pseudo-kinetic methods. The penalization method was discussed in more detail in the previous section. The equilibrium equation is implemented in the conservation equations by penalization on the system to be solved. The penalty is applied by a sufficiently large magnitude penalty constant for the ionic balance to be

respected. The penalization is performed on the deviation from thermodynamic equilibrium through the ionic equilibrium equation (Eq. (S.14)) using a penalization constant k; we retrieve the following system,

$$\int \sum_{i=1}^{N} \delta c_i \left[\frac{\partial c_i}{\partial t} + \nabla \cdot \left(-D_i c_i - z_i c_i F u_i \nabla \phi_{liq} \right) - R_i - k \psi_i s_i \left[K - \frac{\prod_{i=1}^{n} a_i^{s_i}}{\prod_{i=n+1}^{m} a_i^{s_i}} \right] \right] dV = 0 \qquad \text{(S.22)}$$

$$\int \delta \phi_{liq} \nabla \cdot \left[F \sum_{i=1}^{N} z_i \left(-D_i c_i - z_i c_i F u_i \nabla \phi_{liq} \right) \right] dV = 0 \qquad \text{(S.23)}$$

The penalization method for implementing equilibrium in a system allows for applying Neumann or Dirichlet-type boundary conditions on the variable ci without eliminating or substituting the conservation equation of the species l involved in the equilibrium. However, unlike the other methods presented, this method does not reduce the number of equations and variables to be solved, which is its main disadvantage.

4. Evaluation of Ionic Equilibrium Methods

A conductivity problem in an aqueous medium with ion balance has been defined to jointly compare ion equilibrium and electroneutrality implantation methods. The studied system is based on an ionic equilibrium in a dilute aqueous solution based on the reaction of EDTA (Y^{-4}) forming a complex with the ferrous ion, and the reaction writes as follows,

$$Fe^{+2} + Y^{-4} \rightleftharpoons FeY^{-2} \qquad \text{(S.24)}$$

EDTA makes several complexes possible, but only one is investigated to simplify the problem. Therefore, pH effects are also neglected. The activities of the ions are assumed to be equal to their concentrations. The ion balance Eq. (S.24) is represented by Eq. (S.25),

$$K = \frac{a_{FeY^{-2}}}{a_{Fe^{+2}}} \cong \frac{c_{FeY^{-2}}}{c_{Fe^{+2}} c_{Y^{-4}}} \qquad \text{(S.25)}$$

The problem to be solved is a simple transient ionic conduction problem involving ionic equilibrium. The aqueous solution is initially composed of $FeCl_2$, NaCl, and Na_4Y salts dissociating completely. Ion concentrations balance according to the ionic reaction (Eq. (S.24)) to respect the equilibrium condition described by Eq. (S.25). All the ions making up the system (Na^+, $Fe^{+2,}$ Cl^-, $Y^{-4,}$ and FeY^{-2}) can carry charges. The solution is considered dilute and stagnant, so convection is negligible.

The current imposed at the boundary Γ_1 is constant and produces a molar flux of Fe^{+2} similar to an anodic electrochemical reaction having no charge transfer overvoltage. So, the boundary Γ_1 is closed to other ions. At the Γ_2 border, Dirichlet-type conditions are imposed. The system does not allow a free net charge, so it must remain macroscopically electrically neutral.

The equilibrium constant is arbitrary but approximately of the order of magnitude of certain equilibrium constants associated with EDTA (Butler et al. [6]], and the diffusion coefficients for the ions associated with EDTA are of the same order of magnitude as the real ones [7]. Table S1 presents the solution's constituents before applying the initial ionic balance, diffusion coefficients, and other parameters. The mobilities are obtained from the Nernst-Einstein equation (Eq. (S.5)). The stoichiometry s_i and the parameter Ψ_i are not used for the elimination method. Similarly, the parameter k is used only for the

penalization method. The value of the parameter k was not optimized but sufficiently high to ensure the respect of the ionic balance for a relative error in an interval of ± 0.5%.

Table S1. Parameters of study case of conduction in aqueous medium with ionic equilibrium.

**Diffusion coefficients and species' parameters**							
i	z_i	s_i	Ψ_i	D_i			
Na^+	+1	0	0	$1.33x10^{-9}m^2s^{-1}$			
Cl^-	-1	0	0	$2.03x10^{-9}m^2s^{-1}$			
Fe^{+2}	+2	1	-1	$0.719x10^{-9}m^2s^{-1}$			
FeY^{-2}	-2	1	1	$0.4x10^{-9}m^2s^{-1}$			
Y^{-4}	-4	1	-1	$0.5x10^{-9}m^2s^{-1}$			
Composition of aqueous solution before application of initial ionic equilibrium and other parameters							
C_{Nacl}	C_{FeCl_2}	C_{Na_4F}	T	K	k	i_0	n
10^{-2}mol m^{-3}	10^{-3}mol m^{-3}	10^{-3}mol m^{-3}	298.15 K	100	$1x10^{12}$	0.02 A.m^{-2}	2

Table S2 provides the initial and boundary conditions for the problem, but some of these conditions cannot be applied using methods that eliminate species conservation equations. Additionally, the Lagrange variable is not included in all methods for implementing ionic equilibrium and electroneutrality, as it can be seen by examining the equations of the various techniques described below.

Table S2. Initial and boundary conditions of condition problem in aqueous medium with ionic equilibrium ($x \in [0, L]$, L = 0.001m).

**Boundary Conditions**	
$C_{Na^+}(x,0)$	$0.014\ molm^{-3}$
$C_{Cl^-}(x,0)$	$0.012\ molm^{-3}$
$C_{Fe^{+2}}(x,0)$	$9.160798\times10^{-4}\ molm^{-3}$
$C_{Y^{-4}}(x,0)$	$9.160798\times10^{-4}\ molm^{-3}$
$C_{FeY^{-2}}(x,0)$	$8.392022\times10^{-5}\ molm^{-3}$
$\Phi(x,0)$	0V
$\lambda(x,0)$	1
**Boundary Conditions**	
Γ_1	Γ_2
$-\vec{n}_1\cdot\vec{J}_{Na^+}=0\ mol\cdot m^{-2}\cdot s^{-1}$	$C_{Na+}(L,t)=0.014\ mol\cdot m^{-3}$
$-\vec{n}_1\cdot\vec{J}_{Cl^-}=0\ mol\cdot m^{-2}\cdot s^{-1}$	$C_{Cl-}(L,t)=0.012\ mol\cdot m^{-3}$
$-\vec{n}_1\cdot\vec{J}_{Fe^{2+}}=\dfrac{i_0}{nF}$	$C_{Fe^{2+}}(L,t)=9.160798\times10^{-4}\ mol\cdot m^{-3}$

$-\vec{n}\cdot\vec{J}_{Y^{-4}}=0\ mol\cdot m^{-2}\cdot s^{-1}$	$C_{Y^{-4}}(L,t)=9.160798\times10^{-4}\ mol\cdot m^{-3}$
$-\vec{n}\cdot\vec{J}_{FeY^{-2}}=0\ mol\cdot m^{-2}s^{-1}$	$C_{FeY^{-2}}(L,t)=8.392022\times10^{-5}\ mol\cdot m^{-3}$
$-\vec{n}\times\vec{i}=0.02\ A\times m^{-2}$	$\Phi(L,t)=0\text{V}$
	$\lambda(L,t)=1$

Several methods were combined and tested to put into perspective the effect of the different resolution methods on the solution. Table S3summarizes these methods. In the PenElim method, electroneutrality was used to estimate the C_{Na+} concentration posterior. Its effect has been considered in the conservation of charges, but the PDE corresponding to the conservation equation has been crossed out. The PenMulti case includes the Na^+ ion conservation PDE, and electroneutrality is satisfied using a Lagrange multiplier to impose this constraint. The details of the formulations are given below. The PenElim method of ion balance implantation is the method using penalization. The application of electroneutrality in the case of the PenElim method is carried out according to the elimination method.
The elimination of the Na^+ ion conservation equation applies electroneutrality. If we apply the Eqs. (S.2), (S.3), and (S.4) for the problem stated in this section, one obtains the following system of equations to be solved through,

$$\int\sum_i \delta c_i\left[\frac{\partial c_i}{\partial t}+\nabla\cdot\left(-D_i\nabla c_i-z_ic_iFu_i\nabla\phi_{liq}\right)-k\psi_is_i\left[K-\frac{c_{FeY^{-2}}}{c_{Fe^{2+}}c_{Y^{-4}}}\right]\right]dV=0 \tag{S.26}$$

i = Cl^-,Fe^{2+},Y^{-4},FeY^{-2}

$$\int\delta\phi_{liq}\nabla\cdot\left[F\sum_i z_i\left(-D_i\nabla c_i-z_ic_iFu_i\nabla\phi_{liq}\right)\right]dV=0 \tag{S.27}$$

i = Na^+,Cl^-,Fe^{2+},Y^{-4},FeY^{2-}

$$c_{Na^+}=\frac{-1}{z_{Na^+}}\sum_i z_ic_i\quad i = Cl^-, Fe^{2+}, Y^{-4}, FeY^{2-} \tag{S.28}$$

Subsequently, the definition of the variable c_{Na+} from Eq. (S.28) is substituted into Eq. (S.27). This last system, which is made up of five equations and variables (c_{Na+}, c_{F2+}, c_{cl}^-, c_{Y-4}, λ, Φ), is solved numerically. The PenMulti method uses the same ion equilibrium elimination method as the PenElim method, but electroneutrality is applied by the method with a multiplier. Therefore, the Na+ ion conservation equation is not eliminated. The system of equations to be solved is as follows,

$$\int\sum_i \delta c_i\left[\frac{\partial c_i}{\partial t}+\nabla\cdot\left(-D_i\nabla c_i-z_ic_iFu_i\nabla\phi_{liq}\right)-k\psi_is_i\left[K-\frac{c_{FeY^{2-}}}{c_{Fe^{2+}}c_{Y^{-4}}}\right]+z_i\lambda\right]dV=0 \tag{S.29}$$

i = Na^+,Cl^-, Fe^{2+},Y^{-4},FeY^{2-}

$$\int\delta\phi_{liq}\nabla\cdot\left[F\sum_i z_i\left(-D_ic_i-z_ic_iFu_i\nabla\phi_{liq}\right)\right]dV=0 \tag{S.30}$$

i = Na^+,Cl^-,Fe^{2+},Y^{-4},FeY^{2-}

$$\int \delta\lambda \left(\sum_i z_i c_i \right) dV = 0,\ i = \text{Na}^+, \text{Cl}^-, \text{Fe}^{2+}, \text{Y}^{-4}, \text{FeY}^{2-} \qquad \text{(S.31)}$$

The system, therefore, consists of seven equations and seven variables (c_{Na+}, c_F^{2+}, c_{cl-}, c_{Y-4}, λ, Φ). Table S3 summarizes the ion equilibrium and electroneutrality implantation methods investigated. Table S4 shows the various parameters used in COMSOL 6.0 Multiphysics for the numerical resolution by FEM. The mesh and the various parameters used to resolve the equations systems are the same in all the given methods.

Table S3. Methods used in this study case.

**Methods**	Ionic Equilibrium			Electroneutrality	
	Elimination	Source Term	Penalization	Elimination	Lagrange Multiplier
PenElim			X	X	
PenMulti			X		X

Table S4. Resolution parameters used in COMSOL Multiphysics 6.0 for the numerical simulations with FEM.

Parameters	Values
Consistent initialization of PDE-algebraic system	Backward Euler
Implicit resolution method with variable order and variable time step	BDF (Backward differentiation formula)
Time step	$t \in [0{,}100\text{s}]$
Direct linear solver	UMFPACK
Constraints treatment method	Elimination
Relative tolerance	10^{-3}
Absolute tolerance	10^{-4}
Meshing in Comsol 6.0 Multiphysics	Free mesh
Element growth rate	1.4
Maximum element size	1x10-6 m
Maximum element dimension scale factor	1
Application mode	General form of PDE
Methods	DOF
PenElim	5015
PenMulti	7037

Figures S1-(a) and (b) depict the concentration of Cl^- and Fe^{+2} ions at 100 seconds for the different methods. For these last two ions, the methods give similar results, which do not necessarily make it possible to distinguish whether they are equivalent or not clearly. Figure S1-(c) demonstrates a relative constancy of the potential compared to the different methods, suggesting that they are electrically equivalent. This observation can be corroborated by the fact that none of the methods eliminates transport parameters at the level of the charge conservation equation.

It should also be noted that the conservation equations for the Cl^- and Fe^{+2} ions are never eliminated or substituted in the methods studied, which means that the transport parameters are always present in all the methods and not only in the charge conservation equation.

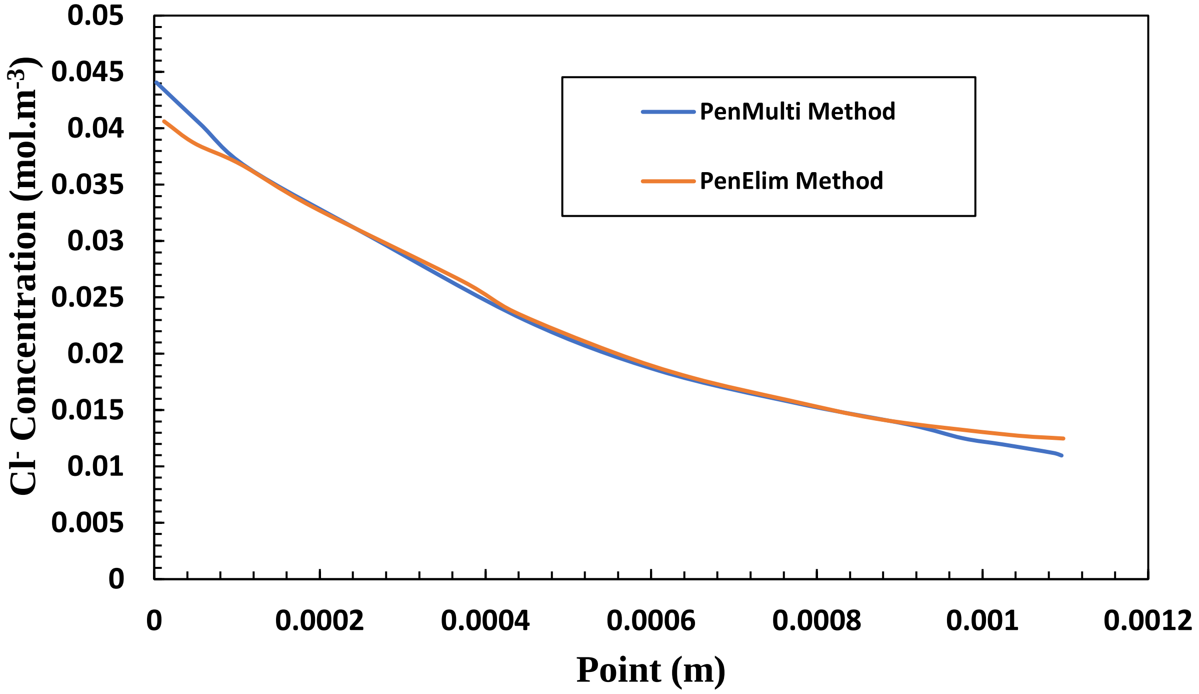

(a)

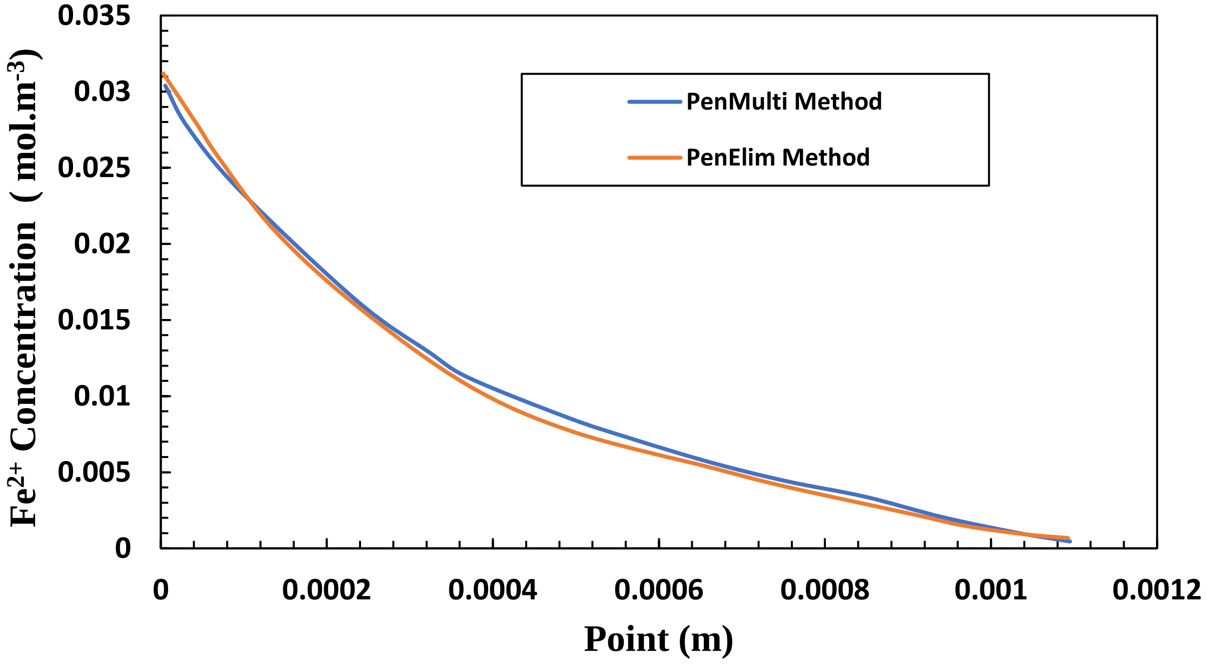

(b)

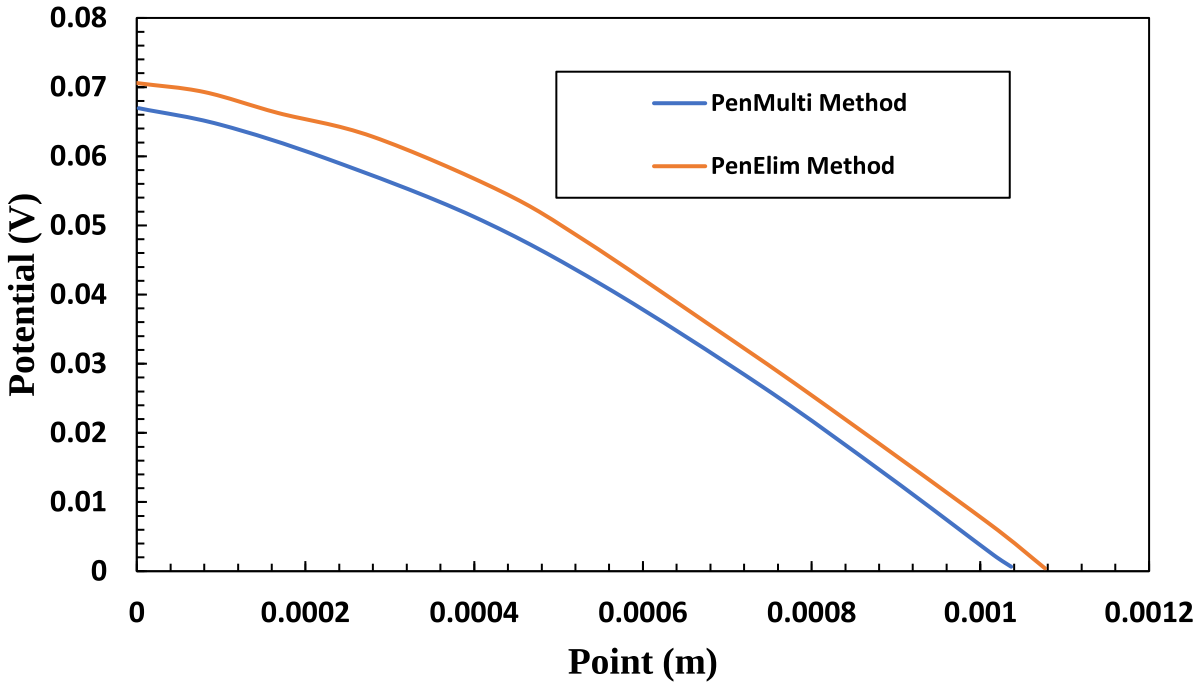

(c)

Fig.S1: Evolution of species concentration in the aqueous solution as the function of the point at time 100 seconds calculated through PenElim and PenMulti methods: (a) Cl^- species; (b) Fe^{2+} and (c) the potential.

Figure S2-(a) shows the concentration of the Y^{-4} ion at 100 seconds for the different methods. Figure S1-(a) shows that ways that retain the conservation equation for the Y^{-4} ion by penalization or substitution through the source term yield nearly identical results. Electroneutrality by Lagrange multiplier also gives similar curves regardless of the ion balance implantation procedure. All the methods have a similar aspect, i.e., the tendency to form a depression towards the center. In this study, the Y^{-4} ion is the only other species whose conservation equation is never eliminated, whatever the method used in this problem. Figure S2-(a) clearly shows the equivalence between the method of penalization and substitution. The curves of these methods remain similar for the same method of implementing electroneutrality. Figure S2-(b) shows similar results for the FeY^{-2} ion, i.e., the ion equilibrium implantation methods give a similar curve for the same electroneutrality implantation method. In Figure S2-(b), the penalization and substitution method equivalence are even more discernible.

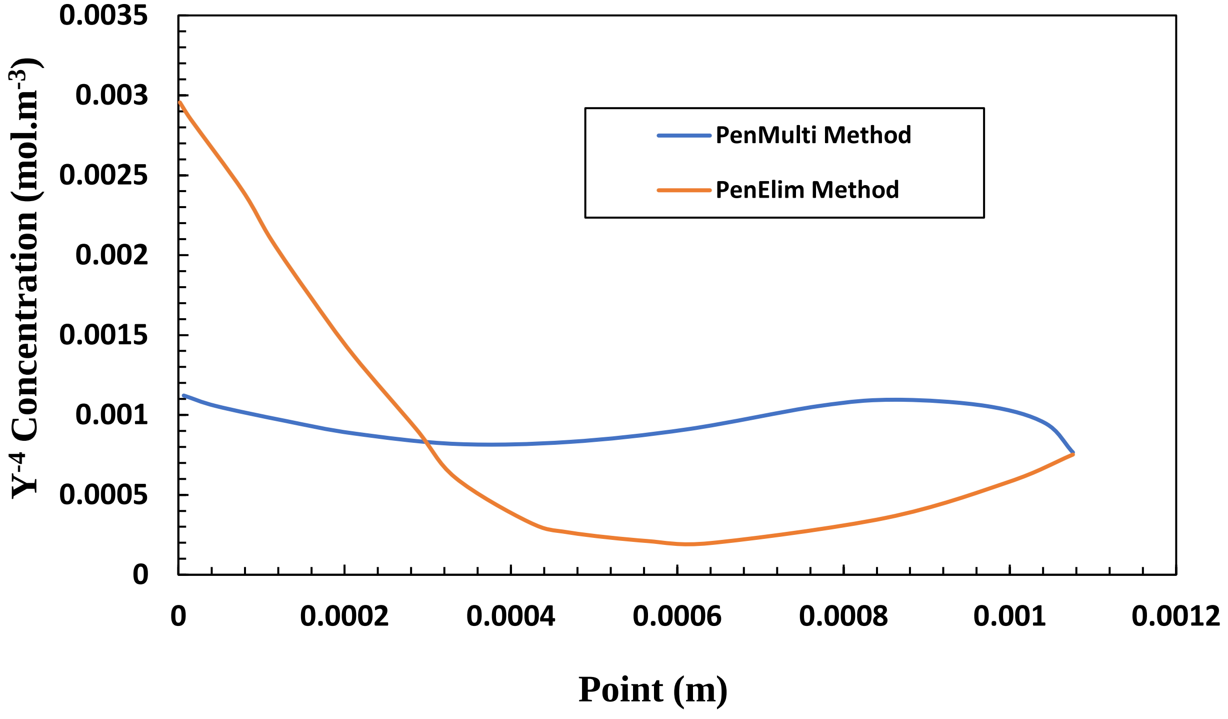

0.0035
0.003
0.0025
0.002
0.0015
0.001
0.0005
0
0
0.0002
0.0004
0.0006
0.0008
0.001
0.0012
Point (m)
Y^{-4} Concentration (mol.m^{-3})
PenMulti Method
PenElim Method

(a)

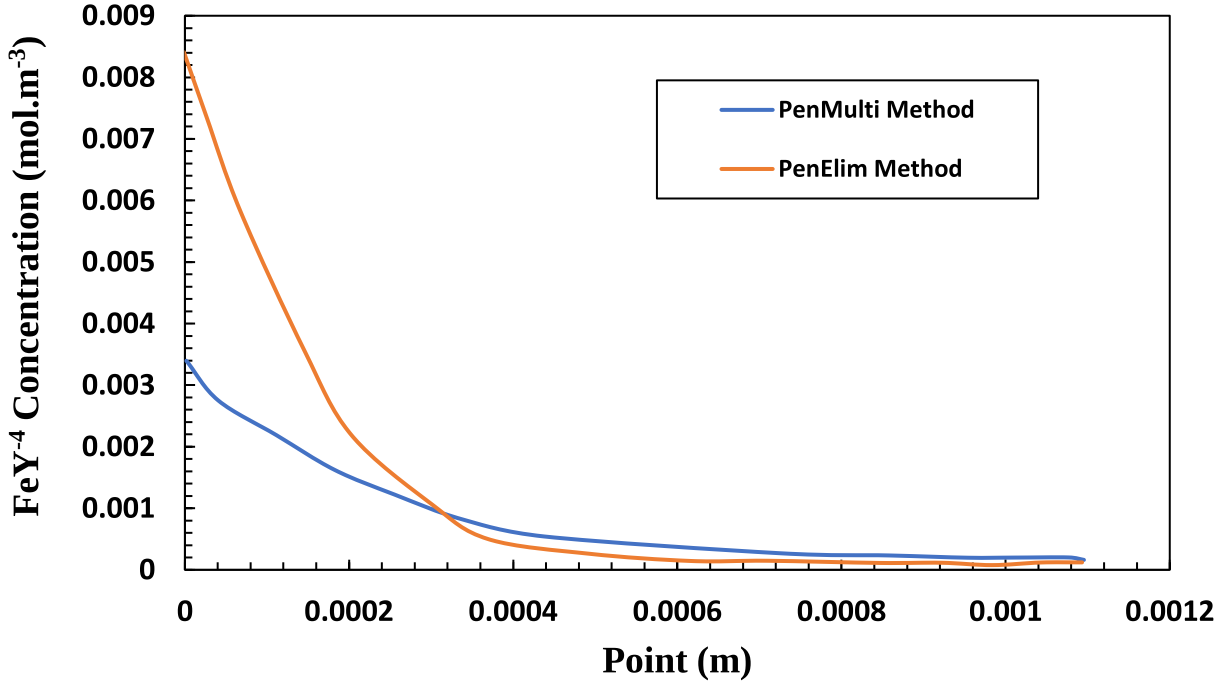

0.009
0.008
0.007
0.006
0.005
0.004
0.003
0.002
0.001
0
0
0.0002
0.0004
0.0006
0.0008
0.001
0.0012
Point (m)
FeY^{-4} Concentration (mol.m^{-3})
PenMulti Method
PenElim Method

(b)

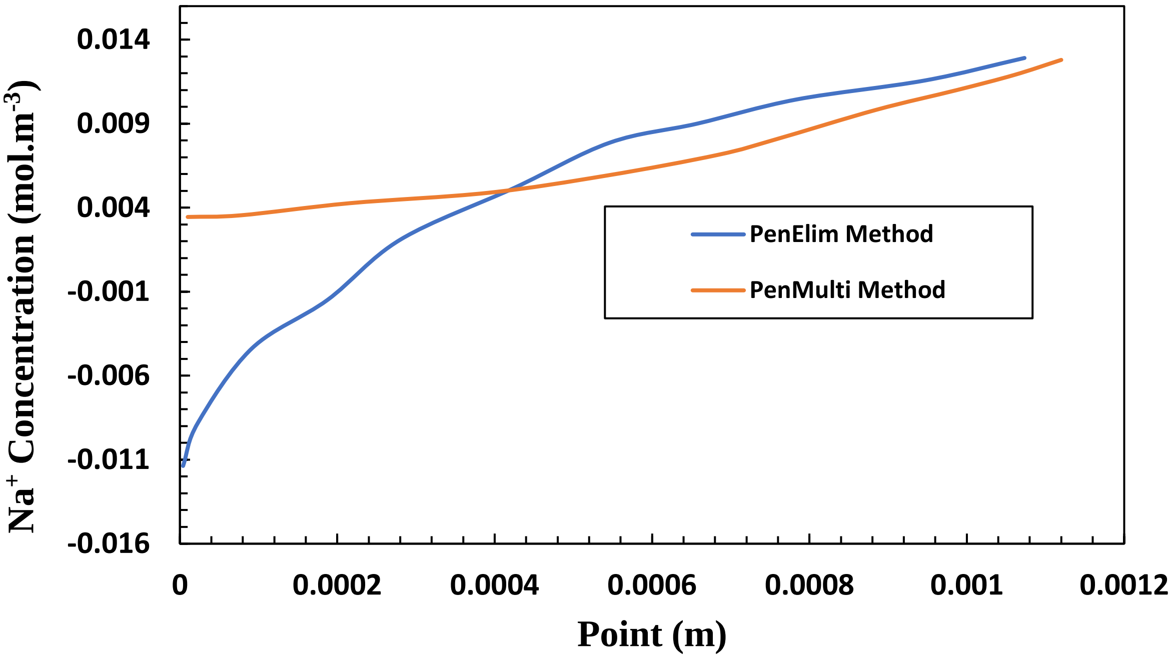

(c)

Fig. S2. Evolution of species concentration in the aqueous solution with ionic equilibrium as a function of the point at time t =100 seconds calculated through PenElim and PenMulti methods: (a) species $Y^{-4;}$ (b) species FeY^{-2}; (c) Na+ specie.

Figure S2- (c) shows that the PenElim method produces negative concentrations for the ion Na^{+}. This last observation shows that eliminating a species conservation equation to respect electroneutrality can lead to numerical errors that increase with time, especially if the concentration values approach zero. The absence of Na^{+} ion transport and the presence of the conservation equation for FeY^{-2} perturbs the system. Table S5 shows that the methods perform relatively well compared to each other in terms of electroneutrality and respect for ionic balance. The PenElim method curve, despite presenting a negative concentration of $Na^{+,}$ still performs well in electroneutrality, emphasizing that other factors beyond electroneutrality must be examined when evaluating a model's performance. This last point shows that electroneutrality is not the only criterion to be considered in the analysis of the performance of a model.

Table S5 Verifying electroneutrality and respect of ionic balance in the 100 s resolution domain.

**Methods**	**Relative error on ionic equilibrium:** $100\left[K-\dfrac{C_{FeY^{-2}}}{C_{Fe^{+2}}C_{Y^{-4}}}\right]\Big/K$	**Electroneutrality:** $\sum_{i=1}^{5} z_i c_i$
PenElim	$[0, 0.187\%]$	$\pm 5.24\times10^{-18}$
PenMulti	$\pm 1\times10^{-3}\%$	$\pm 2.4\times10^{-17}$

References

1. J. Newman and W. Tiedemann, Porous-electrode theory with battery applications, AlChe Journal 21(1), pp.25-42, 1975.https://doi.10.1002/aic.690210103.
2. A. Fortin, Analyse numérique pour ingénieurs, Editions de l'école polytechnique de Montréal, pp.448-454,3rd Edition, Presses internationales polytechnique,1995.
3. M.W. Saaltink, C.Ayora, J. Carrera, A mathematical formulation for reactive transport that eliminates mineral concentrations, Water Resources Research 34(7), pp.1649-1656,1998. https://doi:10.1029/ 98 WR00552.
4. C.I.Steefel, A.C.Lasagna, A coupled model for transport of multiple chemical species and kinetic precipitation/dissolution reactions with applications to reactive flow in a single-phase hydrothermal systems, American Journal of Science 294 (5), pp.529-592, 1994.https://doi.org/10.2475/ajs.294. 5.529.
5. K.T.B. MacQuarrie, K.U.Mayer, Reactive transport modeling in fractured rock: A state-of-the-science review, Earth-Science Reviews 72(3-4), pp.189-227, 2005.https://doi.org/10.1016/j. earscirev.2005. 07. 003.
6. J.N.Butler and D.R.Cogley, Ionic equilibrium (solubility and pH calculations), John Wiley and Sons Inc., New-York, USA, pp.559-573,1998.
7. D.G.Leaist and L.Hao, Tracer diffusion of some metal ions and Metal-EDTA complexes in aqueous sodium chloride solutions, Journal of the Chemical Society Faraday Transactions 90(1), pp.133-136, 136, 1996.https://doi.org/10.1039/FT9949000133.
